# Fimbriatols A–J, Highly Oxidized *ent*-Kaurane Dit*e*rpenoids from Traditional Chinese Plant *Flickingeria fimbriata* (B1.) Hawkes

**DOI:** 10.1038/srep30560

**Published:** 2016-08-03

**Authors:** Gang Ding, Jiaodong Fei, Jing Wang, Yong Xie, Rongtao Li, Ningbo Gong, Yang Lv, Changyuan Yu, Zhongmei Zou

**Affiliations:** 1 Key Laboratory of Bioactive Substances and Resources Utilization of Chinese Herbal Medicine, Ministry of Education, Institute of Medicinal Plant Development, Chinese Academy of Medical Sciences and Peking Union Medical College, Beijing 100193, P. R. China; 2 Beijing University of Chemical Technology, Beijing 100029, People’s Republic of China; 3 Hainan Branch of Institute of Medicinal Plant Development, Chinese Academy of Medical Sciences and Peking Union Medical College, Beijing 100193, People’s Republic of China; 4 Institute of Materia Medica, Chinese Academy of Medical Sciences and Peking Union Medical College, Beijing 100050, People’s Republic of China

## Abstract

Fimbriatols A–J (1–10), ten new *ent*-kaurane diterpenoids possessing differently highly oxidized sites, were isolated from *Flickingeria fimbriata* (B1.) Hawkes. The structures of these new compounds were determined by HRESI-MS, NMR, CD spectra and X-ray diffraction analysis. Compound 1 displayed moderately inhibitory ratio (48.5%) compared with the positive compound NSC-87877 (81.6%) at the concentration of 0.022 *μ*g/mL. Compounds 7–10 possess 3, 4-seco-*ent*-kaurane skeleton containing a disaccharide moiety with an unusual linkage at C-2′ to C-1′′ instead of the common linkage at C-6′ to C-1′′, and this is the first report in 600 more *ent*-kauranes found in nature, which might be originated from *ent*-kaurane diterpenoids through post-modified reactions of Baeyer-Villiger oxygenation and glycosylation.

*Ent*-kaurane diterpenoids are a big member of terpenoids with diverse structural features. More than 600 *ent*-kaurane diterpenoids have been isolated from different plants, especially from the genus *Isodon* (=*Rabdosia*)^1^,^2^,^3^. Recently, some novel *ent*-kaurane diterpenoids such as neoadenoloside A^4^, neolaxiflorins A and B^5^ with unique skeletons were reported, which led to a new wave of research hot about *ent*-kaurane diterpenoids. According to the structure features, Professor Fujita ever divided this member of diterpenoids into four groups: *ent*-kauranes, 6,7-seco-*ent*-kauranes, 8,9-*seco*-*ent*-kauranes, and others in 1984^6^. Recently Professor Sun classified *ent*-kauranes into nine groups including C-20 non-oxygenated ones, C-20 oxygenated ones, 6,7-seco-*ent*-kauranes, 8,9-seco-*ent*-kauranes, 8,15-seco-*ent*-kauranes, 15,16-seco-*ent*-kauranes, 7,20-seco-*ent*-kauranes, *ent*-kaurane dimers, miscellaneous *ent*-kauranes^1^. In addition, there were also some reports about 2, 3-seco-*ent*-kauranes (Fig. 1)^7^. Although 3, 4-seco-sesquiterpenoids, and 3, 4-seco-triterpenoids were ever isolated from different sources, 3, 4-seco- *ent*-kaurane diterpenoids have never found in nature (Fig. 1).

*Flickingeria fimbriata* (B1.) Hawkes is a medicinal plant distributed in the south of China including Hainan, Guangxi, and Yunnan Provinces. Its whole herb has been used as tonic and antipyretic agents in the Minority Nation Li, in Hainan province. In addition, this medicinal plant (also named “You Gua Shi Hu”) was often used as the equivalent of *Pale Ephemerantha* (“Shi Hu”) in the medicinal market. Previous chemical investigations of this plant obtained pimarane-type diterpenoids, several phenanthrenes, steroids, and bibenzyls^7^,^8^,^9^,^10^,^11^,^12^. During our ongoing search of new bioactive secondary metabolites from herbal medicines^13^,^14^,^15^, we initiated the chemical study of this medicinal plant collected from Hainan province. Ten new *ent*-kaurane diterpenoids fimbriatols A–J (**1**–**10**) including four 3, 4-seco-*ent*-kauranes (**7**–**10**) were isolated (Fig. 2). Herein we reported the isolation, structural elucidation, bioactive evaluation and postulated biosynthesis of 3, 4 *ent*-kaurane diterpenoids.

## Results and Discussion

**Figure 3:**
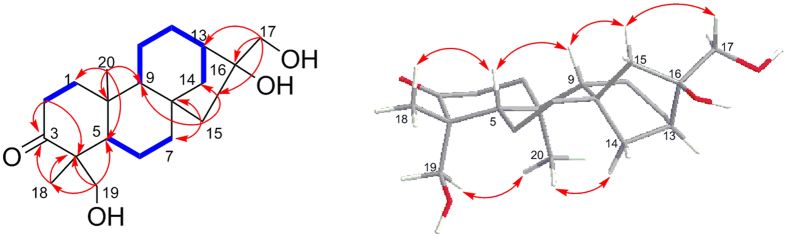
Selective 2D NMR correlations of 1.

Compound **1** had molecular formula C_20_H_32_O_4_ on the basis of its HRESIMS (*m*/*z* 337.2380 [M + H]^+^, Δ − 0.1) indicating the presence of five degrees of unsaturation. Detailed analysis of the ^1^HNMR, ^13^CNMR, and HMQC data for **1** (Tables 1 and 2) revealed the presence of two methyls, ten methylene units (two oxygenated), three methines, and four quaternary carbons (one oxygenated), and one ketone group. These data accounted for all ^1^H and ^13^C resonances except for three exchangeable protons, and required **1** to be four cyclic systems. Interpretation of the ^1^H–^1^H COSY NMR data led to the identification of three isolated proton spin-systems corresponding to the C-1–C-2, C-5–C-6–C-7, and C-9–C-11–C-12–C-13–C-14 fragments of structure **1**. The remaining connectivity was determined by HMBC correlations (Fig. 3). The correlations from CH_3_-18 and OCH_2_-19 to C-3, C-4, and C-5 confirmed that C-4 was connected with C-3, C-5, C-18 and C-19, and correlations from CH_2_-1 to C-3, and CH_2_-2 to C-3 and C-4 implied the keto group C-3 was connected with C-2 and C-4. Those correlations from CH_3_-20 to C-1, C-5, C-9, and C-10 revealed that C-10 was connected with C-1, C-5, C-9 and C-20, whereas the correlations of CH_2_-15 with C-7, C-8, C-9, and C-14 confirmed that C-7, C-9, C-14 and C-15 all were connected with C-8. In the HMBC spectra, the distinct cross peaks from OCH_2_-17 to C-13, C-15, and C-16 confirmed the connectivity of C-16 with C-13, C-15 and C-17. Accounting for the molecular formula and chemical shift values of C-16, C-17 and C-19, it implied that these three carbons possessed hydroxyl groups, respectively. Thus the planar structure of **1** was characterized. SciFinder searching found a phyllocladane diterpenoid named 16,17,18-trihydroxyphyllocladan-3-one possessing the same planar structure as that of **1**^16^. Liu *et al*. ever summarized the diagnostic^13^ C NMR chemical shifts of selected phyllocladanes and *ent*-kauranes, which revealed that chemical shift values of of C-13, C-14, C-15, C-16, C-17 and C-20 in these two member of diterpenoids changed regularly^17^. From the^13^ C NMR chemical shifts of those carbons in compound **1**, it implied that this structure should possess the skeleton of *ent*-kauranes not that of phyllocladanes. This hypothesis was further confirmed by NOESY correlations and CD spectra. The correlations from H_2_-17 to H-15a, H-15a to H-9, H-9 to H-5, H-5 to CH_3_-18 implied the *β* configuration of these protons, whereas the correlations of CH_3_-20 with H_2_-14 and OCH_2_-19 established the *α* configuration of these protons (Fig. 3), which established the relative configuration of compound **1** as kauranes. The absolute configuration was determined by CD spectra (Supporting Information), which displayed the negative conton effects at 240, 290 nm, and positive one at 320 nm opposite to (16*R*)-16,17-dihydroxy-phyllocladan-3-one. This result confirmed that the stereo-center of C-5 was *S*-configuration. Thus the absolute configuration of compound **1** was determined to 4*R*, 5*S*, 8*S*, 13*R*, 16*R*^17^, which revealed that compound **1** indeed possessed the skeleton of *ent*-kauranes.

**Figure 4:**
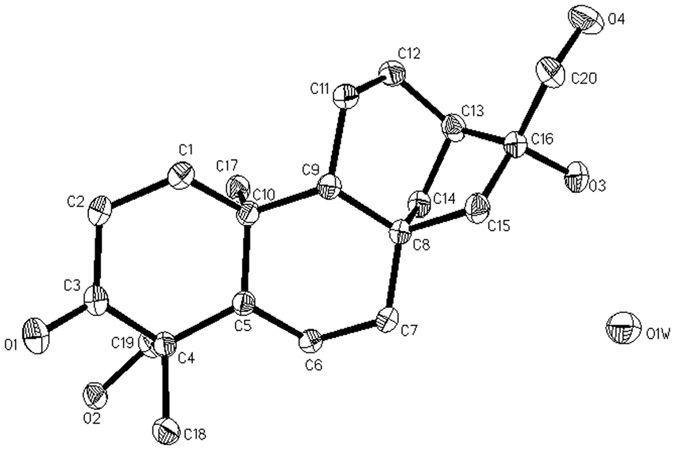
ORTEP diagram of 1.

Fortunately, suitable crystal of **1** was obtained for X-ray crystallography, and the Flack factor was -0.1, which determined the stereochemistry of **1** as (4*R*, 16*R*)-16, 17, 19- trihydroxy-*ent*-kaur-3-one (Fig. 4).

**Figure 5:**
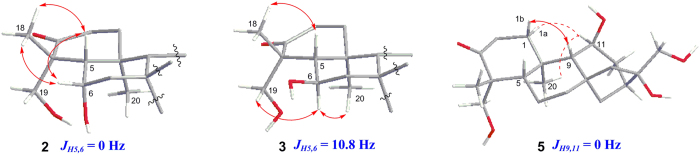
Coupling constant analysis and Key NOESY correlations of 2, 3 and 5.

The HRESIMS of **2** gave a pseudomolecular ion [M + Na]^+^ peak at *m/z* 375.2061 (Δ+8.6), indicating the molecular formula of **2** as C_20_H_32_O_5_ (five degrees of unsaturation) with one more oxygen atom than that of **1**. Analysis of NMR data revealed one additional oxygenated methine (*δ*_C_ 65.86; *δ*_H_ 4.29) present in NMR spectra in **2** compared with that of **1**. The ^1^H–^1^H COSY and HMBC correlations confirmed that the CH_2_-6 in **1** was oxygenated to the corresponding oxymethine unit in **2**. The relative configuration was established by analysis of coupling constant and NOESY correlations. The small coupling constant between H-5 and H-6 (*J* ≈ 0 Hz) together with the NOESY correlations from H-5, H-6 to CH_3_-18 implied the *cis* relationship of this two protons. The other NOESY correlations were same as those of **1** (Fig. 5). Thus the relative configuration of **2** was determined.

The molecular formula of **3** was same as that of **2** by analysis of its HRESIMS. The NMR data especially the 2D spectra revealed that **3** possessed the same planar structure as **2**. The NOESY correlation spectrum of **3** was similar with that of **2**, except that H-6 had correlations with H_2_-19 and CH_3_-20, not correlation with CH_3_-18 in **2**, leading to the *trans* configuration between H-5 and H-6, and this was also supported by the big coupling constant between H-5 and H-6 (*J* = 10.8) (Fig. 5).

Compound **4** possessed the same as molecular formula as that of **2** and **3** on the basis of HRESIMS (m/z 375.2169 [M + Na]^+^, Δ − 2.7) implying that **4** was an isomer of **2** and **3**. The NMR data mainly including the 2D NNR experiments suggested that the hydroxyl group was attached at C-9 not at C-6 found in **2** and **3**. The NOESY correlation revealed the same relative configuration as those of **1**–**3** except for the 9-OH.

The HRESIMS of **5** suggested the same molecular formula as compounds **2**–**4**. The NMR spectra revealed the similar structure feature as those present in **1** except the additional hydroxyl group was connected at C-11, which was confirmed by H–^1^H COSY and HMBC correlations. The small coupling constant (*J* ≈ 0 Hz) between H-9 (singlet) and H-11 implied that the dihedral angel was 90°. The NOESY correlations from H-11 with CH_3_-20, and H-1a revealed that the 11-OH possessed the *β*-configuration (Fig. 5).

The HRESI MS (m/z 375.2134 [M + Na]^+^ Δ + 0.8) of compound **6** afforded the molecular formula as C_20_H_32_O_5_ same as that of compounds **2**–**5**. The ^1^H NMR spectra displayed three methyl groups present in **6**, implying that the 19-methyl was not oxidized. The ^1^H–^1^H COSY and HMBC correlations confirmed that both C-1 and C-6 contained one hydroxyl group, respectively. The hydroxyl group for C-1 was determined to be *α*-configuration on the basis of the NOESY correlations from H-1 to H-5 and H-9, and hydroxyl group at C-6 was established as *β*-configuration on the basis of NOESY correlations of H-6 with CH_3_-19 and CH_3_-20. In addition, the large coupling constant between H-5 and H-6 (*J* ≈ 10.8 Hz) supported the *trans* relationship of these two protons.

Compounds **2–6** displayed the similar CD spectra with that of compound **1**, which implied the same stereochemistry of **2–6** as that of **1** (Supporting Information).

**Figure 6:**
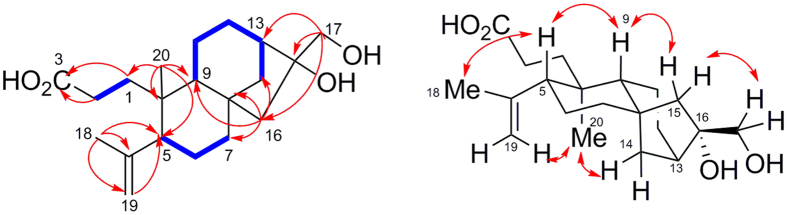
Key HMBC and NOESY correlations of 7.

The molecular formula of **7** was determined to be C_20_H_32_O_4_ on the basis of HRESI MS *m*/*z* 359.2181 ([M + Na]^+^). Analysis of the ^1^H and ^13^C NMR data of **7** revealed the significant difference compared with those of **1** (Table 3). The NMR spectra displayed a carboxylic group (*δ*_H_ 11.94; *δ*_C_ 174.7) and a double of exocyclic olefinic carbons (*δ*_H_ 4.83, 4.62; *δ*_C_ 147.1, 113.1) signals, whereas the keto carbonyl (C-3) and the oxymethylene (C-19) were disappeared. These differences implied that the carbon-bond of C-3/C-4 might be oxidized to shape the corresponding carboxylic group, and then dehydration reaction at C-19 formed the exocyclic double bond. The 2D NMR experiments especially the HMBC correlations from CH_2_-1 and CH_2_-2 to C-3 (*δ*_C_ 174.7) and from CH_3_-18 to C-4, C-5 and C-19 (exocyclic olefinic carbon) confirmed the above-mentioned postulation (Fig. 6). Thus the planar structure of **7** was determined. The relative configuration was established by NOESY correlations. Compound **7** possessed the same relative configuration as that of **1** depicted in Fig. 6.

Compound **8** possessed the molecular formula as C_25_H_40_O_4_ on the basis of HREIMS (*m/z* 404.2903 [M]^+^, Δ + 2.4). Comparison of the NMR data of compound **8** and **7** revealed that the same structural fragments were found in **8** except for an additional oxyethyl and an acetonide group present in **8** (Table 3). In addition, the carboxylic acid group, and the two hydroxyls at C-16 and C-17 were disappeared. This difference implied that the diol group at C-16/17 might react with acetone to form the acetonide product, and the carboxyl group might shape the oxyethyl derivative with ethanol solvent, and this postulation was further supported by HMBC correlations. Because ethanol and acetone solvents were used in the process of isolation, compound **8** was postulated to be an artifact product from **7.** We added **7** to acetone and ethanol solvent mixed with silica gel for a week. The mixture was then analyzed by TLC, which did produce compound **8** by comparison. This revealed that compound **8** should be the artifact product from **7**.

The molecular formula of **9** was established as C_31_H_50_O_13_ by analysis of its HRESIMS [*m/z* 653.3179 (M + Na)^+^; Δ − 3.0 mmu] and NMR data (Table 3). The ^1^H and ^13^C NMR spectra for **9** suggested the presence of the same structure fragment found in **7** and **8** except for two additional sugar moieties. The HMQC, ^1^H–^1^H COSY and HMBC correlations confirmed the two sugar moieties were glucose and apiose, respectively. The coupling constant of the anomeric proton in the glucose moiety (*δ* 4.37, 1H, d, *J* = 7.8 Hz) suggested a *β*-configured glucose unit, whereas the coupling constant for the apiose anomeric proton (*δ* 5.20, 1H, d, *J* = 3.0 Hz) and the chemical shift value for the anomeric carbon signal at *δ* 109.9 suggested the *β*-configured apiose unit. The linkage of the sugar moieties was determined by HMBC correlations. The HMBCcorrelations from CH_2_-17 to C-1′, in turn, from H-1′ to C-17 implied the connection of C-17 with C-1′ by an oxygen atom. The linkage between C-2′ and C-1′′ by an ether bond was confirmed by HMBC correlations from H-1′′ to C-2′, and from H-2′ to C-1′′. The chemical shifts of the glucose and apiose moiety were assigned through ^1^H–^1^H COSY, HMQC and HMBC spectra. Thus the planar structure of **9** was established. The relative configuration of **9** was same as that of **7** by analysis of the NOESY correlations.

The molecular formula of **10** was established as C_32_H_52_O_13_ by analysis of its HRESIMS [*m/z* 667.33214 (M + Na)^+^; Δ  − 1.4 mmu] with 14 mass units more than that of compound **9**. The ^1^H and ^13^C NMR spectra of **10** displayed resonances nearly identical to those of **9** except for an additional OMe group, suggesting that **10** was the oxymethyl derivative of **9**. Analysis of HMBC data confirmed the above observations, and permitted the OMe unit to be connected with the carboxyl group (C-3). The relative configuration of **10** was same as those of **7** and **9** by analysis of its NOESY correlations. Due to the different groups found in compounds **7–10**, the CD spectra of compunds **7–10** were different from those compounds **1–6** (supporting information), which could not determine the absolute configurations of **7–10** by CD spectra. From the biosynthetic view, these compounds were originated from same diterpene biogenetic pathway. Thus compunds **7–10** were postulated to possess the same absolute configurations as those **1–6**. The configurations of two sugar moieties were determined to be D-glucose and D-apiose by hydrolysis and GC-MS methods^18^.

Protein phosphorylation as posttranslational modification plays a vital role to regulate cell activities. In cells, the protein kinases and phosphatases control the protein phoshorylation level^19^. Dual-specificity phosphatase 26 (DUSP26) is a heterogeneous group of protein phosphatases, and can dephosphorylate both phosphotyrosine and phosphoserine/phosphothreonine residues^20^. Due to the fact that DUSP26 is located at 8p, a chromosomal region that has been shown to be amplified in anaplastic thyroid cancer (ATC), this enzyme has emerged as a potential target for the treatment of human cancers by dephosphorylating p38 MAPK, thereby inhibiting p38-mediated apoptosis^21^. Only one compound, 8-hydroxy-7-(6-sulfonaphthalen-2-yl)diazenyl-quinoline-5-sulfonic acid (NSC-87877), had been found as a DUSP26 inhibitor with the against DUSP26 IC_50_ value of 18.6 μM^19^, however, up to date no research was reported about natural products inhibiting against DUSP26. Thus compound **1** were tested inhibitory activities against DUSP26, and displayed biological activity with inhibitory ratio at 48.5% compared with 81.6% of the positive compound NSC-87877. Though the bioactive result of compound **1** was not good as the positive compound, the skeleton feature of **1** is completely different from that of the positive compound NSC-87877, which might imply their possibly different site of action.

In addition, compounds **1**–**10** were also tested against the activities of several plant pathogens, acetylcholinesterase, xanthinoxidase (XOD) and cytotoxic activities without any effects.

**Figure 7:**
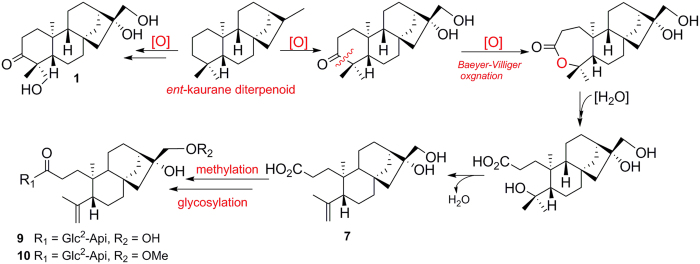
Postulated biogenetic pathway of 1, 7, 9 and 10.

Up to date, more than 600 *ent*-kaurane diterpenoids with diverse structurally features have been isolated from different plant^1^,^2^,^3^. Compounds **1**–**6** are different from all known analogues by possessing highly oxidized sites, whereas **7**–**10** contain the 3, 4-seco-*ent*-kaurane skeleton, which is the first report in all *ent*-kaurane diterpenoids. Mono-glycosylated *ent*-kaurane diterpenoids are ever isolated from different resource, whereas **9** and **10** contained a disaccharide moiety at C-17 with the unusual linkage at C-2′ to C-1′′ not the common linkage at C-6′ to C-1′′, which was the first report in all known *ent*-kaurane diterpenoids. Recently, Hu *et al*. confirmed that a flavin-dependent monooxygenase catalyzed a keto group to the corresponding ester moiety by Baeyer-Villiger reaction in *Aspergillus clavatus*^21^. Thus we postulated that compounds **7**–**10** might be biosynthesized from *ent*-kaurane diterpenoids firstly through Baeyer-Villiger oxidation reaction to cleave the carbon bond between C-3 and C-4, and then through hydration, dehydration and glycosylation reactions to form **9** and **10** by post-modification (Fig. 7). Considering the highly oxidized sites in compounds **1**–**10**, it implies that there exist diverse P450 oxidases or flavin-dependent monooxygenases in this plant, which could produce different *ent*-kaurane diterpenoids analogues, and thus further chemical investigation of this medicinal plant should be in progress to mine more new *ent*-kaurane diterpenoids. In conclusion, ten new *ent*-kaurane diterpenoids possessing highly oxidized sites were isolated from *Flickingeria fimbriata* (B1.) Hawkes. Our result further diversifies the structural feature of *ent*-kaurane diterpenoids.

## Methods

### General experimental procedures

Optical rotations were measured on a Perkin-Elmer 241 polarimeter, and UV data were recorded on Beckman Coulter DU 800 spectrometer. IR data were recorded using a Shimadzu FTIR-8400S spectrophotometer. ^1^H and ^13^C NMR data were acquired with a Bruker 600 spectrometer using solvent signals (CDCl_3_; *δ*_H_ 7.26/*δ*_C_ 77.6, CD_3_OD; *δ*_H_ 3.31/*δ*_C_ 49.9, DMSO-*d*_6;_ *δ*_H_ 2.49/*δ*_C_ 39.5) as references. The HMQC and HMBC experiments were optimized for 145.0 and 8.0 Hz, respectively. HRESIMS data were acquired using a LTQ Orbitrap XL mass spectrometer.

### Plant material

The plant of *Flickingeria fimbriata* (B1.) Hawkes was collected from Changjiang, Hainan Province, People’s Republic of China, in August 2008. The sample was identified by Mr. Rong-tao, Li (Hainan Branch of Institute of Medicinal Plant Development,), and a voucher specimen (FF2008-1) has been deposited in the herbarium of the Institute of Medicinal Plant Development, Chinese Academy of Medical Sciences, Beijing.

### Extraction and isolation

The air-dried and smashed stems of *F. fimbriata* were extracted with 95% EtOH to afford a crude extract after evaporation under vacuum. The extract was suspended in H_2_O and then partitioned sequentially with petroleum ether, CH_2_Cl_2_, EtOAc, and *n*-BuOH, successively. The CH_2_Cl_2_ and EtOAc extracts were subjected to column chromatography over silica gel to obtain 10 new *ent*-kaurane diterpenoids named fimbriatols A–J (**1**–**10**), which were characterized by HRESIMS, NMR and X-ray diffraction experiments.

The air-dried and smashed stems of *F. fimbriata* (3.5 kg) were extracted with 95% EtOH (3 × 14 L) to afford a crude extract (330 g) after evaporation under vacuum. The extract was suspended in H_2_O (2.0 L) and then partitioned sequentially with petroleum ether (3 × 3.0 L), CH_2_Cl_2_ (3 × 3.0 L), EtOAc (3 × 3.0 L), and *n*-BuOH (3 × 3.0 L), successively. The CH_2_Cl_2_ extract (80.0 g) was subjected to column chromatography over silica gel (80–100 mesh), eluted with petroleum ether-acetone (10:1~1:1) as the mobile phase to yield eight fractions (C1~C8). E4 (3.2g) was subjected to column chromatography over silica gel (100–200 mesh) repeatedly to obtain **8** (16 mg). E6 (18.0 g) eluted with petroleum ether-acetone (3:2) was subjected to column chromatography over silica gel (100–200 mesh), eluted with ether-acetone (50:1~4:1) repeatedly, and then recrystallization to afford compounds **1** (3.0 g) and **7** (15 mg), respectively. E7 (18.0 g) eluted with petroleum ether-acetone (1:1) was subjected to column chromatography over silica gel (100–200 mesh), Sephadex LH-20, ODS and HW-40C (ethanol: H_2_O = 1:1) to afford compounds **2** (2.0 mg), **3** (2.0 mg), and **5** (10.0 mg), respectively. The EtOAc extract (10.0 g) was subjected to column chromatography over silica gel (100–200 mesh), eluted with CH_2_Cl_2_-CH_3_OH (50:1~3:1) to yield eight fractions (E1~E7). E6 (1.0 g) was subjected to column chromatography over ODS and HW-40C to **10** (14.6 mg). E7 (1.5 g) was subjected to column chromatography over silica gel (100–200 mesh), then Sephadex LH-20, ODS and HW-40C to **9** (73.0 mg). E7 (6.0 g) eluted with petroleum ether-acetone (1:1) was subjected to column chromatography over silica gel (100–200 mesh), eluted with ether-acetone (7:3~0:1) and Sephadex LH 20 column chromatography to obtain **4** (5.0 mg) and **6** (7.5 mg), respectively.

Fimbriatol A (4*R*, 16*R*)-16, 17, 19- trihydroxy-*ent*-kaur-3-one (**1**): white crystal; mp 194–196 °C; [α]^20^_D_ -20.0 (*c* 0.06, MeOH); UV (MeOH) λ_max_ (log ε) 205 (0.5), 282.5 (0.027) nm; IR (KBr) *ν*_max_ 3372 (OH), 2928 (CH), 1693 (C=O) cm^−1^; ^1^H and ^13^C NMR see Tables 1 and 2; HRESI-MS *m*/*z* 337.2380 [M + H]^+^ (calcd for C_20_H_33_O_4_, 337.2379).

Fimbriatol B (4*R*, 6*S*, 16*R*)-6, 16, 17, 19- tetrahydroxy-*ent*-kaur-3-one (**2**): white crystal; mp 220–222 °C. 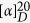 −10.0(*c* 0.05, MeOH); UV (MeOH) λ_max_ (log ε) 210 (0.16), 292.5 (0.018) nm; IR (KBr) *ν*_max_3200 (OH), 2922 (CH), 1696 (C = O) cm^−1^; ^1^H and ^13^C NMR see Tables 1 and **2**; HRESI-MS *m*/*z* 375.2061 [M + Na]^+^ (calcd for C_20_H_32_O_5_Na,375.2142).

Fimbriatol C (4*R*, 6*R*, 16*R*)-6, 16, 17, 19- tetrahydroxy-*ent*-kaur-3-one (**3**): white crystal; mp. 226–228 °C; 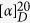
−38.0 (*c* 0.075, MeOH); UV (CH_3_OH) λ_max_ (log ε) 210 (0.30) nm; IR (KBr) *ν*_max_ 3381 (OH), 2931(CH), 1696 (C=O) cm^−1^; ^1^H and ^13^C NMR see Table 1 and 2; HRESI MS (+) *m/z* 375.2164 [M + Na]^+^ (calcd for C_20_H_32_O_5_Na,375.2142).

Fimbriatol D (4*R*, 16*R*)-9, 16, 17, 19- tetrahydroxy-*ent*-kaur-3-one (**4**): white crystal; mp. 116–118 °C; 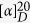 –11.4 (*c* 0.07, MeOH); UV (CH_3_OH) λ_max_(log ε) 212 (0.26) nm; IR (KBr) *ν*_max_ 3367 (OH), 2945(CH), 1685 (C=O) cm^−1^; ^1^H and ^13^C NMR see Tables 1 and 2; HRESIMS(+) *m/z* 375.2169 [M + Na]^+^ (calcd for C_20_H_32_O_5_Na,375.2142).

Fimbriatol E (4*R*, 11*S*, 16*R*)-11, 16, 17, 19- tetrahydroxy-*ent*-kaur-3-one (**5**): white crystal; mp. 202–204 oC; 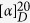 −16.7 (*c* 0.06,MeOH); UV (CH_3_OH) λ_max_(log ε) 212 (0.26), 282.5 (0.015) nm; IR (KBr) *ν*_max_ 3382 (OH), 2931 (CH), 1694 (C=O) cm^−1^; ^1^H and ^13^C NMR see Tables 1 and 2; HRESIMS (+) *m/z* 375.2167 [M + Na]^+^ (calcd for C_20_H_32_O_5_Na,375.2142).

Fimbriatol F (1*S*, 6*R*, 16*R*)-1, 6, 16, 17- tetrahydroxy-*ent*-kaur-3-one (**6**): amorphous powder; mp. 284–286 ^o^C; 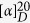 −59.1 (*c* 0.055,MeOH); UV (CH_3_OH) λ_max_(log ε) 206 (0.27), 285 (0.022) nm; IR (KBr) *ν*_max_ 3378 (OH), 2928 (CH), 1708 (C=O) cm^−1^; ^1^H and ^13^C NMR see Tables 1 and 2; HRESIMS(+) *m/z* 375.2134 [M + Na]^+^ (calcd for C_20_H_32_O_5_Na,375.2142).

Fimbriatol G (**7**): white crystal; mp 236–238 °C; [α]^20^_D_ -68.3 (*c* 0.01, MeOH); UV (CH_3_OH) λ_max_(log ε) 204 (1.48), 237 (0.021) nm; IR (KBr) *ν*_max_ 3437 (OH), 2925(CH), 1706 (C=O) cm^−1^; ^1^H and ^13^C NMR data see Table 3; HRESI-MS *m/z* 359.2198 [M + Na]^+^ (calcd for C_20_H_32_O_4_Na, 359.2198).

Fimbriatol H (**8**): white crystal; mp 200-202 °C; [α]^20^_D_ -19.5 (*c* 0.02, MeOH); UV (CH_3_OH) λ_max_(log ε) 205 (5.0) nm; IR (KBr) *ν*_max_ 2926(CH), 1734 (C = O) cm^−1^; ^1^H and ^13^C NMR see Table 3; HREIMS (*m/z* 404.2903 [M]^+^ (calcd for C_25_H_40_O_4_, 404.2927).

Fimbriatol I (**9**): brown oil; [α]^20^_D_ - 80 (*c* 0.01, MeOH); UV (CH_3_OH)λ_max_ (log ε) 202 (1.2) nm; IR (KBr) *ν*_max_ 3401 (OH), 2929 (CH), 1705 (C = O) cm^−1^; ^1^H and ^13^C NMR see Tabled **3**; HRESI-MS *m/z* 653.3179 (M + Na)^+^ (calcd for C_31_H_50_O_13_Na, 653.3149).

Fimbriatol J (**10**): brown oil; [α]^20^_D_ - 66 (*c* 0.01, MeOH); UV (CH_3_OH)λ_max_ (log ε) 202 (1.263) nm; IR (KBr) *ν*_max_ 3376 (OH), 2929 (CH), 1730 (C = O) cm^−1^; ^1^H and ^13^C NMR see Table 3; HRESI-MS *m*/*z* 667.3328 (M + Na)^+^ (calcd for C_32_H_52_O_13_Na, 667.3307).

### Hydrolysis reaction and GC-MS analysis

Compounds **9** and **10** (1mg each) were put in 1 N HCl (0.25 mL) and stirred at 80 °C for 4 h. Then after cooling, the solution was dried by blowing air^18^. The residue was dissolved in 1-(trimethylsilyl)-imidazole and pyridine (1 mL). The solution was stirred at 60 °C for 5 min. After drying the solution with a stream of air, the residue was separated by water and CH_2_Cl_2_ (1 mL, v/v = 1:1). The CH_2_Cl_2_ layer was analyzed by GC (Agilent technologies 7890B system) using an capillary HP-5 column (30 m*250 μm*0.25 μm). Temperature was maintained at 50 °C for 1min, then raised to 100 °C at the rate of 5 °C/min (5 min), and raised to 265 °C at the rate of 5 °C/min (11 min). Peaks of the hydrolysate of **9** and **10** were detected at 15.25, 15.32, 16.49 min, and 15.25, 15.32, 16.49 min, respectively. Peaks of D-apiose, were 15.27, 16.49 min, and D- and L-glucose were 15.24, and 15.30 min, respectively.

### X-ray crystallographic analysis of fimbriatone (1)

Crystallization from petroleum MeOH: H_2_O (10: 1) yielded colorless prisms of **1.** A crystal (0.08 × 0.14 × 0.52mm) was separated from the sample and mounted on a glass fiber, and data were acquired with a a Rigaku MicroMax 002 + diffractometer with graphite-monochromated Mo Kα radiation and a graphite monochromator. Structure analysis was made using the SHELXL97 program. Crystallographic data (excluding structure factors) for **1** in this paper have been deposited with the Cambridge Crystallographic Data Centre (deposition number CCDC 1063873). Copies of the data can be obtained, free of charge, on application to CCDC, 12 Union Road, Cambridge CB2 1EZ, UK (fax: + 44 12 23336033 or e-mail: deposit@ccdc.cam.ac.uk). Crystal data: C_20_H_32_O_4_·H_2_O, M = 336.47, space group P2_1_2_1_2_1_; unit cell dimensions a = 6.083(3), b = 11.417(3), c = 26.629(6)Å, monoclinic, *V* = 1849.4(12)Å^3^, *D*_*c*_ = 1.273g/cm^3^, *Z* = 4. A total of 592 maps and 3145 independent reflections were collected in the range of 0° < Θ < 180°, of which 2995 observable reflections [|F|^2^>2σ|F|^2^)], completeness to Θ max was 96.7%; non-hydrogen atoms were refined anisotropically. Hydrogen atoms were located in Fourier difference maps and refined with idealized geometries and riding constraints. The final indices were R_1_ = 0.0377, wR_2_ = 0.0977, S = 1.049.

### *In vitro* enzyme-based assay

#### DUSP26 expression and purification

The DNA fragment encoding DUSP26 was subcloned from human DUSP26 cDNA (Invitrogen, Carlsbad, CA, USA) into the expression vector pGEX-4T-2 as a fusion with a N-terminal GST-tag and a thrombin protease cleavage site. For proteins expression, *Escherichia coli* cells growing in 1 l LB media at OD_600_ of near 0.8 were inducted with 0.5 mM IPTG at 16 °C for 16 h. Centrifuged culture pellets were extracted by sonication in PBS buffer with 100 mM PMSF. Extracts were clarified by centrifugation 13,000 rpm for 30 min at 4 °C to yield the soluble extract. GST-tagged proteins present in the soluble extracts were purified by GST-affinity chromatography and dialyzed overnight against buffer containing 50 mM Tris–HCl (pH 7.5), and 0.1 M NaCl.

#### Assays of DUSP26 inhibition

*In vitro* phosphatase assay used for determining whether compounds are DUSP26 inhibitors was established and optimized based on methods described previously^22^,^23^. The activity of DUSP26 was measured using the substrate p-nitrophenyl phosphate (pNPP; Sigma, St. Louis, MO) at the concentration using a 96-well microtiter plate. NSC-87877 (positive inhibitor; Calbiochem, San Diego, CA) and pNPP were solubilized in ddH_2_O. The compound **1** were solubilized in HPLC grade DMSO with concentration at 0.022 μg/mL. All reactions were performed at a final concentration of 1% DMSO, while the enzyme activity was not affected by this concentration of DMSO.

The purified DUSP26 (10 nM), and a candidate inhibitor were incubated in the reaction mixture containing 50 mM Tris–HCl (pH 7.5), 1% DMSO, for 15 min at 37 °C. Reactions were initiated by addition of pNPP and the incubation time was 30 min at 37 °C. This enzymatic reaction was stopped with the addition of 0.2 M NaOH. The phosphatase activities were then monitored by measuring the absorbance changes due to the hydrolysis of the substrate at 405 nM. The activities of DUSP26 with and without candidate inhibitors were represented as Ai and A0. The value of Ai/A0 × 100% was the residual activity (R.A.) of DUSP26. All experiments were performed in triplicate.

## Additional Information

**How to cite this article**: Ding, G. *et al*. Fimbriatols A-J, Highly Oxidized *ent*-Kaurane Diterpenoids from Traditional Chinese Plant *Flickingeria fimbriata* (B1.) Hawkes. *Sci. Rep.* **6**, 30560; doi: 10.1038/srep30560 (2016).

## Supplementary Material

Supplementary Information

## Figures and Tables

**Figure 1:**
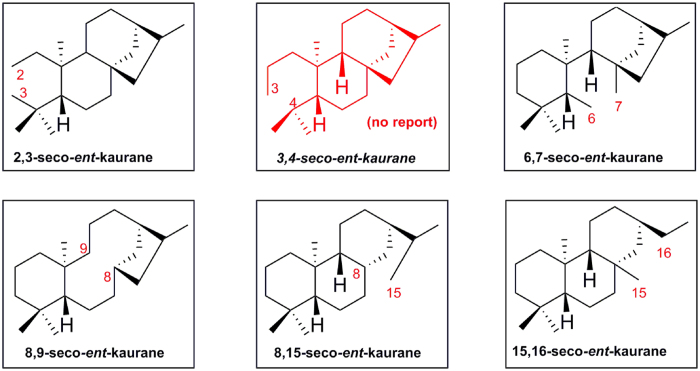
Different types of seco-*ent*-kaurane diterpenoids.

**Figure 2:**
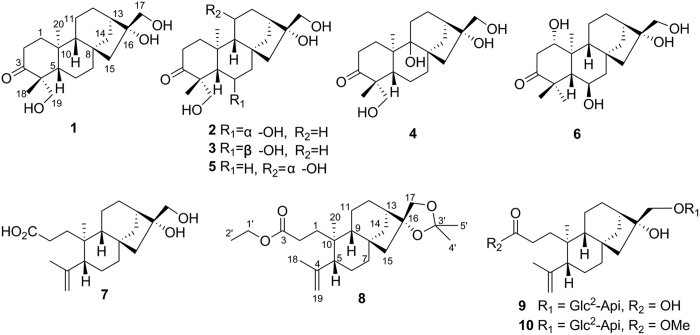
Compounds 1–10 isolated from *Flickingeria fimbriata* (B1.) Hawkes.

**Table 1:** ^1^H NMR data of compounds 1–6 recorded at 600 Hz.

Pos	1^a^	2^a^	3^b^	4^a^	5^a^	6^a^
1a 1b	2.13, m 1.33, m	2.05, m 1.32, m	1.81, m 1.47, m	2.31, m 1.77, m	2.22, ddd (3.6, 6.6, 13.2) 1.60, m	3.92, dd (1.2, 9.0)
2	2.69, m 2.33, m	2.60, m 2.37, m	2.72, ddd (5.4, 11.4, 15.6) 2.12, ddd (5.4, 9.6, 15.6)	2.67, ddd (6.6, 11.4, 14.4) 2.39, ddd (4.8, 6.6, 14.4)	2.74,ddd (6.6, 12.6, 15.6) 2.38, ddd 3.0, 5.4, 15.6)	3.28, dd (9.0, 14.4) 2.00, dd (1.8, 14.4)
5	1.42 m	1.33, s	1.64, d (10.8)	2.11, d (11.4)	1.46, d (10.8)	1.58, d (10.8)
6	1.63, 1.53 m	4.29, d (1.8)	3.80, dt (3.6, 10.8)	1.63, m 1.56, m	1.67, m 1.56, m	3.84, dt (3.6, 10.8)
7a 7b	1.66, 1.49 m	1.84, m 1.74, m	1.67, m 1.48, m	1.88, dt (5.4, 13.2) 1.36, m	1.73,m 1.56, m	1.81, dd (4.2, 11.4) 1.61, m
9	1.10 d (6.6)	1.20, br.s	1.06, d (7.2)		1.30, s	1.33, d (7.2)
11a 11b	1.64 m	1.67, m	1.57, m 1.45, m	1.99, dd (7.2, 15.0) 1.30, m	3.93, d (7.2)	2.42, dd, (7.2, 15.6) 1.68, m
12	1.66, 1.56 m	1.79, m 1.63, m	1.54, m 1.31, m	1.79, dd (7.2, 15.0) 1.61, m	2.04, m 1.87, m	1.59, m 1.48, m
13	2.05 br.m	2.06, m	1.89, br.s	2.06, br. s	2.10, br. s	2.03, br. s
14a 14b	1.94, 1.64 m	2.32, d (12.0) 1.83, d (12.0)	1.63, m	2.20, br. d (12.0) 1.79, d (12.0)	1.86, m 1.73, d (10.8)	1.76, m
15a 15b	1.54, 1.40 m	1.61, d (14.4) 1.45, d (14.4)	1.46, m 1.32, d (13.8)	2.27, m 1.09, d (15.0)	2.14, br.d (14.4) 1.34, d (14.4)	1.64, d (11.4) 1.46, d (14.4)
17a 17b	3.70, d (11.4) 3.60, d (11.4)	3.73, d (10.4) 3.63, d (10.4)	3.51, dd (10.8, 3.6) 3.42, dd (10.8, 4.8)	3.69, d (11.4) 3.58, d (11.4)	4.16, d (11.4) 3.76, d (11.4)	3.72, d (11.4) 3.62, d (11.4)
18	1.13, s	1.12, s	1.22, s	1.15, s	1.17, s	1.30, s
19a 19a	3.97, d (11.4) 3.50, d (11.4)	4.13, d (10.4) 3.48, d (10.4)	3.97, d (10.8) 3.73, d (10.8)	4.02, d (11.4) 3.52, d (11.4)	3.97, d (11.4) 3.52, d (11.4)	1.30, s
20	1.24 s	1.49, s	0.82, s	1.36, s	1.17, s	0.93, s
6-OH			5.01, d (3,6)			
16-OH			3.91, s			
17-OH			4.35, dd (4.8, 3.6)			
19-OH			5.11, br.d (3.6)			

^a^Recorded in CD_3_OD, ^b^recorded in DMSO-*d*_6_.

**Table 2:** ^13^C NMR data of compounds 1–6 recorded at 600 Hz.

Pos	1^a^	2^a^	3^b^	4^a^	5^a^	6^a^
1	41.1, CH_2_	41.6, CH_2_	38.1,CH_2_	34.6, CH_2_	40.2,CH_2_	80.1,CH
2	35.9, CH_2_	36.1, CH_2_	32.8, CH_2_	36.1, CH_2_	35.9,CH_2_	46.6,CH_2_
3	218.4, C	217.2, C	217.2, C	219.1, C	218.1,C	220.2,C
4	55.0, C	55.7,C	51.5, C	54.8, C	55.2,C	48.8,C
5	57.9, CH	58.5, CH	58.3, CH	50.2, CH	58.1,CH	56.6,CH
6	22.5, CH_2_	68.5, CH	66.4,CH	22.3, CH_2_	22.4,CH_2_	68.8,CH
7	43.0, CH_2_	48.7, CH_2_	49.6, CH_2_	37.9, CH_2_	43.1,CH_2_	51.6,CH_2_
8	45.5, C	43.6, C	43.8, C	50.8, C	44.0,C	45.8,C
9	57.2, CH	57.0, CH	53.5,CH	78.8, C	66.6,CH	56.3,CH
10	39.9, C	39.6, C	38.7, C	44.5, C	38.8,C	46.5,C
11	19.8, CH_2_	19.9, CH_2_	18.2, CH_2_	30.7, CH_2_	66.3,CH	20.1,CH_2_
12	27.1, CH_2_	27.1, CH_2_	25.3,CH_2_	28.6, CH_2_	37.0,CH_2_	27.0,CH_2_
13	46.3, CH	46.8, CH	44.5, CH	45.2, CH	46.2,CH	46.4,CH
14	38.0, CH_2_	39.8, CH_2_	36.4, CH_2_	39.1, CH_2_	37.2,CH_2_	38.1,CH_2_
15	53.5, CH_2_	54.4, CH_2_	52.5, CH_2_	47.8, CH_2_	51.8,CH_2_	53.4,CH_2_
16	82.7, C	82.1, C	80.0, C	83.0, C	83.1,C	82.5,C
17	66.8, CH_2_	66.9, CH_2_	65.1, CH_2_	66.7, CH_2_	67.3,CH_2_	66.7,CH_2_
18	21.2, CH_3_	22.7, CH_3_	26.0, CH_3_	21.3, CH_3_	21.2,CH_3_	19.6,CH_3_
19	65.5, CH_2_	66.4, CH_2_	62.9, CH_2_	65.7, CH_2_	65.6,CH_2_	32.6,CH_3_
20	18.5, CH_3_	18.6, CH_3_	18.8, CH_3_	21.2, CH_3_	18.3,CH_3_	15.5,CH_3_

^a^Recorded in CD_3_OD, ^b^recorded in DMSO-*d*_6._

**Table 3:** ^1^H NMR and ^13^C NMR spectroscopic data of 7 (in DMSO-*d*_6_), 8 (in CDCl_3_), 9 (in DMSO-*d*_6_) and 10 (in CD_3_OD).

Pos	7		8		9		10	
	*δ*_C,_^b^type	*δ*_H_,^a^mult (*J* in Hz)	*δ*_C,_^b^type	*δ*_H_,^a^mult (*J* in Hz)	*δ*_C,_^b^type	*δ*_H_,^a^mult (*J* in Hz)	*δ*_C,_^b^type	*δ*_H_,^a^mult (*J* in Hz)
1	33.8, CH_2_	1.56, m; 1.43, m	33.9, CH_2_	1.64, m	36.9, CH_2_	1.33, m; 1.53, m	35.3, CH_2_	1.60, m; 1.68, m
2a 2b	28.1, CH_2_	2.17, ddd (4.2, 12.6, 15.6) 1.99, dd (6.0, 12.0)	28.8, CH_2_	2.26, ddd (4.8, 10.8, 16.2) 2.11, ddd (6.6, 11.4, 16.2)	32.3, CH_2_	1.78, m; 1.56, m	29.5, CH_2_	2.16, m 2.32, br.t (12.0)
3	174.7, C		174.0, C		177.7, C		176.4, C	
4	147.1, C		147.3, C		148.2, C		148.8, C	
5	49.2, CH	1.96, dd (2.4, 12.0)	50.2, CH	1.98, dd (2.4, 12.6)	49.5, CH	1.96, d (12.6)	51.4, CH	2.04, br.d (12.6)
6	26.0, CH_2_	1.71, m; 1.31, m	26.3,CH_2_	1.76, m; 1.42, m	26.9,CH_2_	1.70, m; 1.28, m	27.7, CH_2_	1.83, m; 1.42, m
7	40.0, CH_2_	1.42, m	39.8, CH_2_	1.52, m; 1.43, m	40.8, CH_2_	1.40, m	41.4, CH_2_	1.54, m
8	43.5, C		44.1, C		44.1, C		45.4,C	
9	46.0, CH	1.11, d (7.8)	46.3, CH	1.11, d (8.4)	46.3, C	1.19, (6.0)	48.0c, CH	1.20, d (8.4)
10	40.2, C		40.8, C		41.0, C		42.0, C	
11	17.8, CH_2_	1.53, m; 1.38, m	19.0, CH_2_	1.53, m; 1.30, m	18.5, CH_2_	1.50, m	19.4, CH_2_	1.62, m; 1.55, m
12	25.5, CH_2_	1.54, m; 1.36, m	26.87, CH_2_	1.50, m	26.5, CH_2_	1.15, m; 1.36, m	27.3,CH_2_	1.73, m; 1.52, m
13	44.4, CH	1.90, brd (3.0)	45.5, CH	2.16, m	45.5, CH	1.72, m; 1.52, m	46.7, CH	2.12, br.s
14a 14b	36.5,CH_2_	1.74, m 1.57, d (10.8)	38.1, CH_2_	1.93, dd (0.6, 10.8) 1.49, m	37.1, CH_2_	1.62, m; 1.28, m	37.8, CH_2_	1.92, br.d (11.4) 1.66, m
15a 15b	52.8, CH_2_	1.45, m; 1.26, d (14.4)	56.6, CH_2_	1.87, d (14.4) 1.67, d (14.4)	52.9, CH_2_	1.58, m 1.27, m	52.9, CH_2_	1.56, m 1.42, m
16	80.2, C		88.9, C		79.8, C		82.0, C	
17a 17b	65.1, CH_2_	3.51, d (6.0, 10.8) 3.40, d (5.4, 10.8)	69.9, CH_2_	4.05, d (8.4) 3.91, d (8.4)	74.9, CH_2_	3.95, d (9.0) 3.45, d (9.0)	75.1, CH_2_	4.26, d (10.2) 3.46, d (10.2)
18	23.1, CH_3_	1.69, s	23.3, CH_3_	1.73, s	24.4, CH_3_	1.72, s	23.7, CH_3_	1.75, s
19	113.1, CH_2_	4.83, s; 4.62, s	113.6, CH_2_	4.86, s; 4.64, s	113.1, CH_2_	4.82, s; 4.62, s	114.1, CH_2_	4.87, s; 4.67, s
20	21.7, CH_3_	0.92, s	21.9, CH_3_	0.98, s	22.5, CH_3_	0.89, s	23.6, CH_3_	1.02, s
1′/16-OH		4.33, t (5.4)	60.3, CH_2_	4.10, q (7.2)	102.9, CH	4.26, d (7.8)	104.6, CH	4.37, d (7.8)
2′/17-OH		3.86, s	14.2, CH_3_	1.24, t (7.2)	79.7, CH	3.16, dd (9.0, 10.8)	83.8, CH	3.30, m
3′/COOH		11.94, s	108.4, C		76.8, CH	3.32, m	77.5, CH	3.45, m
4′			26.86, CH_3_	1.38 s	70.6, CH	3.10, m	71.5, CH	3.34, m
5′			26.80, CH_3_	1.35 s	77.1, CH	3.10, m	77.8, CH	3.28, m
6′					61.4, CH_2_	3.45, d (11.4) 3.65, d (11.4)	62.7, CH_2_	3.88, d (11.4) 3.67, dd (6.0, 11.4)
1′′					109.7, CH	5.18, d (3.0)	112.3, CH	5.20, d (3.0)
2′′					76.3, CH	3.75, d (3.0)	77.4, CH	3.95, d (3.0)
3′′					79.0, C		79.7, C	
4′′					73.4, CH_2_	3.95, d (9.0) 3.52, d (9.0)	74.6, CH_2_	4.07, d (9.6) 3.73, d (9.6)
5′′					64.2, CH_2_	3.39, d (11.4) 3.34, d (11.4)	65.0, CH_2_	3.57, d (11.4) 3.56, d (11.4)
-OMe							51.4, CH_3_	3.60, s
