# Supplementary material for: Fimbriatols A–J, Highly Oxidized ent-Kaurane Diterpenoids from Traditional Chinese Plant Flickingeria fimbriata (B1.) Hawkes
Source: Sci Rep. 2016 Aug 3;6:30560. doi: 10.1038/srep30560 (PMC4971462; doi:10.1038/srep30560)

Supporting Information

Fimbriatols A–J, Highly Oxidized *ent*-Kaurane Diterpenoids from Traditional Chinese Plant *Flickingeria fimbriata* (B1.) Hawkes†

Gang Ding, ^1^ Jiaodong Fei, ^1,2^ Jing Wang, ^1,2^ Yong Xie,^1^ Rongtao Li,^3^ Ningbo Gong,^4^ Yang Lv,^4^ Changyuan Yu,*^,2^ and Zhongmei Zou*^,1^

**Contents**

1. **Figure S1.** ^1^H NMR spectrum of **1** (600 MHz, CD_3_OD)
2. **Figure S2.** ^13^C NMR spectrum of **1** (150 MHz, CD_3_OD)
3. **Figure S3.** COSY spectrum of **1** (600 MHz, CD_3_OD)
4. **Figure S4.** HMQC spectrum of **1** (600 MHz, CD_3_OD)
5. **Figure S5.** HMBC spectrum of **1** (600 MHz, CD_3_OD)
6. **Figure S6.** NOESY spectrum of **1** (600 MHz, CD_3_OD)
7. **Figure S7.** ^1^H NMR spectrum of **2** (600 MHz, DMSO-*d*_6_)
8. **Figure S8.** ^13^C NMR spectrum of **2** (150 MHz, DMSO-*d*_6_)
9. **Figure S9.** COSY spectrum of **2** (600 MHz, DMSO-*d*_6_)
10. **Figure S10.** HMQC spectrum of **2** (600 MHz, DMSO-*d*_6_)
11. **Figure S11.** HMBC spectrum of **2** (600 MHz, DMSO-*d*_6_)
12. **Figure S12.** NOESY spectrum of **2** (600 MHz, DMSO-*d*_6_)
13. **Figure S13.** ^1^H NMR spectrum of **3** (600 MHz, CD_3_OD)
14. **Figure S14.** ^13^C NMR spectrum of **3** (150 MHz, CD_3_OD)
15. **Figure S15.** COSY spectrum of **3** (600 MHz, CD_3_OD)
16. **Figure S16.** HMQC spectrum of **3** (600 MHz, CD_3_OD)
17. **Figure S17.** HMBC spectrum of **3** (600 MHz, CD_3_OD)
18. **Figure S18.** NOESY spectrum of **3** (600 MHz, CD_3_OD)
19. **Figure S19.** ^1^H NMR spectrum of **4** (600 MHz, CD_3_OD)
20. **Figure S20.** ^13^C NMR spectrum of **4** (150 MHz, CD_3_OD)
21. **Figure S21.** COSY spectrum of **4** (600 MHz, CD_3_OD)
22. **Figure S22.** HMQC spectrum of **4** (600 MHz, CD_3_OD)
23. **Figure S23.** HMBC spectrum of **4** (600 MHz, CD_3_OD)
24. **Figure S24.** NOESY spectrum of **4** (600 MHz, CD_3_OD)
25. **Figure S25.** ^1^H NMR spectrum of **5** (600 MHz, CD_3_OD)
26. **Figure S26.** ^13^C NMR spectrum of **5** (150 MHz, CD_3_OD)
27. **Figure S27.** COSY spectrum of **5** (600 MHz, CD_3_OD)
28. **Figure S28.** HMQC spectrum of **5** (600 MHz, CD_3_OD)
29. **Figure S29.** HMBC spectrum of **5** (600 MHz, CD_3_OD)
30. **Figure S30.** NOESY spectrum of **5** (600 MHz, CD_3_OD)
31. **Figure S31.** ^1^H NMR spectrum of **6** (600 MHz, CD_3_OD)
32. **Figure S32.** ^13^C NMR spectrum of **6** (150 MHz, CD_3_OD)
33. **Figure S33.** COSY spectrum of **6** (600 MHz, CD_3_OD)
34. **Figure S34.** HMQC spectrum of **6** (600 MHz, CD_3_OD)
35. **Figure S35.** HMBC spectrum of **6** (600 MHz, CD_3_OD)
36. **Figure S36.** NOESY spectrum of **6** (600 MHz, CD_3_OD)
37. **Figure S37.** ^1^H NMR spectrum of **7** (600 MHz, DMSO-*d*_6_)
38. **Figure S38.** ^13^C NMR spectrum of **7** (150 MHz, DMSO-*d*_6_)
39. **Figure S39.** COSY spectrum of **7** (600 MHz, DMSO-*d*_6_)
40. **Figure S40.** HMQC spectrum of **7** (600 MHz, DMSO-*d*_6_)
41. **Figure S41.** HMBC spectrum of **7** (600 MHz, DMSO-*d*_6_)
42. **Figure S42.** NOESY spectrum of **7** (600 MHz, DMSO-*d*_6_)
43. **Figure S43.**  ^1^H NMR spectrum of **8** (600 MHz, CDCl_3_)
44. **Figure S44.** ^13^C NMR spectrum of **8** (150 MHz, CDCl_3_)
45. **Figure S45.** COSY spectrum of **8** (600 MHz, CDCl_3_)
46. **Figure S46.** HMQC spectrum of **8** (600 MHz, CDCl_3_)
47. **Figure S47.** HMBC spectrum of **8** (600 MHz, CDCl_3_)
48. **Figure S48.** NOESY spectrum of **8** (600 MHz, CDCl_3_)
49. **Figure S49.** ^1^H NMR spectrum of **9** (600 MHz, DMSO-*d*_6_)
50. **Figure S50.** ^13^C NMR spectrum of **9** (150 MHz, DMSO-*d*_6_)
51. **Figure S51.** COSY spectrum of **9** (600 MHz, DMSO-*d*_6_)
52. **Figure S52.** HMQC spectrum of **9** (600 MHz, DMSO-*d*_6_)
53. **Figure S53.** HMBC spectrum of **9** (600 MHz, DMSO-*d*_6_)
54. **Figure S54.** NOESY spectrum of **9** (600 MHz, DMSO-*d*_6_)
55. **Figure S55.** ^1^H NMR spectrum of **10** (600 MHz, CD_3_OD)
56. **Figure S56.** ^13^C NMR spectrum of **10** (150 MHz, CD_3_OD)
57. **Figure S57.** COSY spectrum of **10** (600 MHz, CD_3_OD)
58. **Figure S58.** HMQC spectrum of **10** (600 MHz, CD_3_OD)
59. **Figure S59.** HMBC spectrum of **10** (600 MHz, CD_3_OD)
60. **Figure S60.** NOESY spectrum of **10** (600 MHz, CD_3_OD)
61. **Figure S61.** X-ray data of compound **1**
62. **Figure S62.** HR-MS of **1-10**
63. **Figure S63** CD spectra of **1-10**

**Figure S1.** ^1^H NMR spectrum of **1** (600 MHz, CD_3_OD)

**Figure S2.** ^13^C NMR spectrum of **1** (150 MHz, CD_3_OD)

**Figure S3.** COSY spectrum of **1** (600 MHz, CD_3_OD)

**
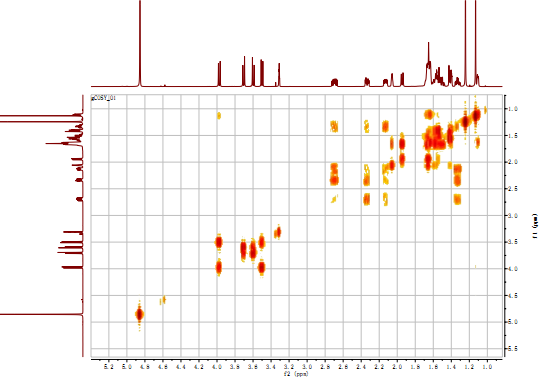
**

**Figure S4.** HMQC spectrum of **1** (600 MHz, CD_3_OD)

**
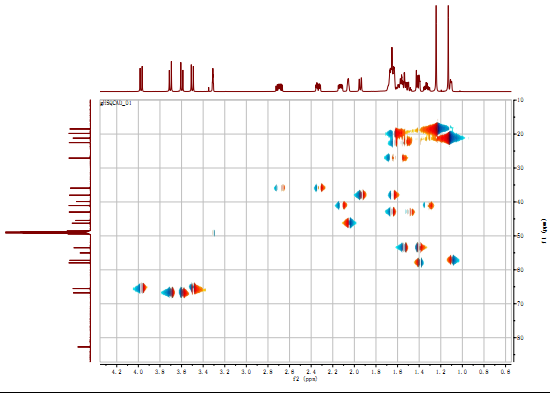
**

**Figure S5.** HMBC spectrum of **1** (600 MHz, CD_3_OD)

**
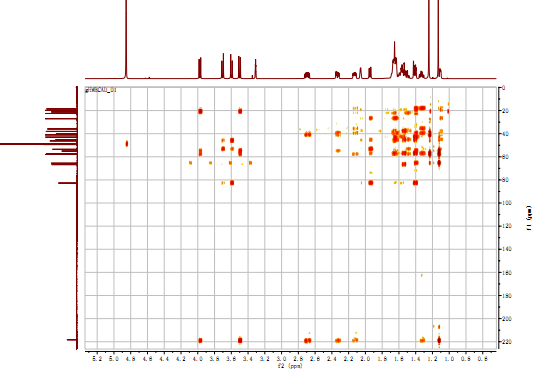
**

**Figure S6.** NOESY spectrum of **1** (600 MHz, CD_3_OD)

**
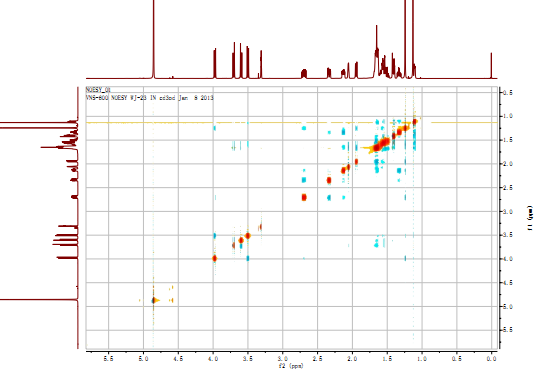
**

**Figure S7.** ^1^H NMR spectrum of (**2**) (600 MHz, DMSO-*d*_6_)

**Figure S8.** ^13^C NMR spectrum of (**2**) (150MHz, DMSO-*d*_6_)

**Figure S9.** COSY spectrum of **2** (600 MHz, DMSO-*d*_6_)


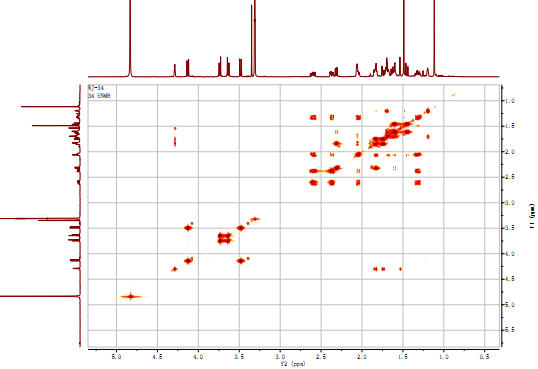


**Figure S10.** HMQC spectrum of **2** (600 MHz, DMSO-*d*_6_)


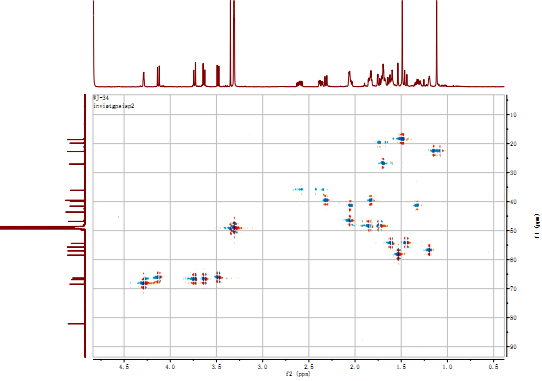


**Figure S11.** HMBC spectrum of **2** (600 MHz, DMSO-*d*_6_)


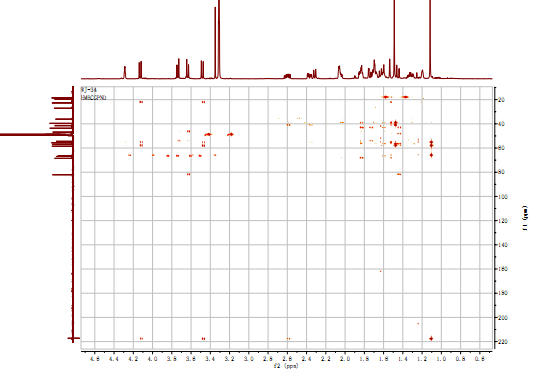


**Figure S12.** NOESY spectrum of **2** (600 MHz, DMSO-*d*_6_)


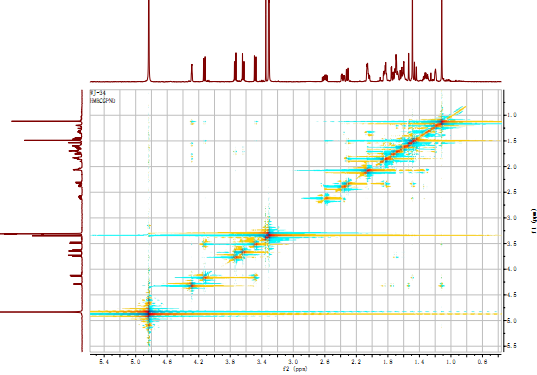


**Figure S13.** ^1^H NMR spectrum of (**3**) (600 MHz, CD_3_OD)

**Figure S14.** ^13^C NMR spectrum of (**3**) (150MHz, CD_3_OD)

**Figure S15.** COSY spectrum of **3** (600 MHz, CD_3_OD)

**Figure S16.** HMQC spectrum of **3** (600 MHz, CD_3_OD)

**Figure S17.** HMBC spectrum of **3** (600 MHz, CD_3_OD)

**Figure S18.** NOESY spectrum of **3** (600 MHz, CD_3_OD)

**Figure S19.** ^1^H NMR spectrum of (**4**) (600 MHz, CD_3_OD)

**Figure S20.** ^13^C NMR spectrum of (**4**) (150MHz, CD_3_OD)

**Figure S21.** COSY spectrum of **4** (600 MHz, CD_3_OD)

**Figure S22.** HMQC spectrum of **4** (600 MHz, CD_3_OD)

**Figure S23.** HMBC spectrum of **4** (600 MHz, CD_3_OD)

**Figure S24.** NOESY spectrum of **4** (600 MHz, CD_3_OD)

**Figure S25.** ^1^H NMR spectrum of (**5**) (600 MHz, CD_3_OD)

**Figure S26.** ^13^C NMR spectrum of (**5**) (150MHz, CD_3_OD)

**Figure S27.** COSY spectrum of **5** (600 MHz, CD_3_OD)

**Figure S28.** HMQC spectrum of **5** (600 MHz, CD_3_OD)

**Figure S29.** HMBC spectrum of **5** (600 MHz, CD_3_OD)

**Figure S30.** NOESY spectrum of **5** (600 MHz, CD_3_OD)

**Figure S31.** ^1^H NMR spectrum of (**6**) (600 MHz, CD_3_OD)

**Figure S32.** ^13^C NMR spectrum of (**6**) (150MHz, CD_3_OD)

**Figure S33.** COSY spectrum of **6** (600 MHz, CD_3_OD)

**Figure S34.** HMQC spectrum of **6** (600 MHz, CD_3_OD)

**Figure S35.** HMBC spectrum of **6** (600 MHz, CD_3_OD)

**Figure S36.** NOESY spectrum of **6** (600 MHz, CD_3_OD)

**Figure S37.** ^1^H NMR spectrum of **7** (600 MHz, DMSO-*d*_6_)

**Figure S38.** ^13^C NMR spectrum of **7** (150 MHz, DMSO-*d*_6_)

**Figure S39.** COSY spectrum of **7** (600 MHz, DMSO-*d*_6_)


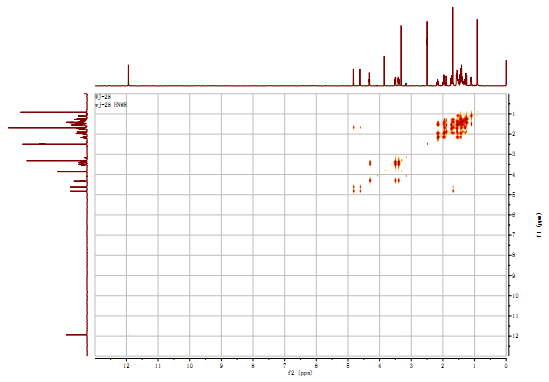


**Figure S40.** HMQC spectrum of **7** (600 MHz, DMSO-*d*_6_)


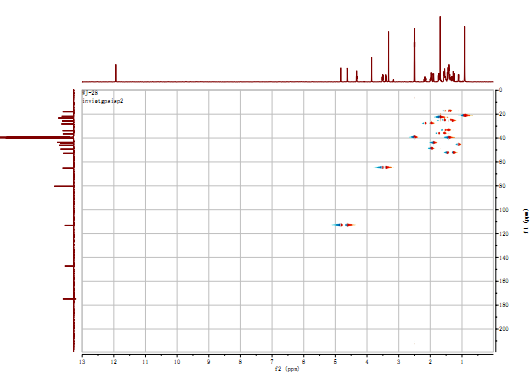


**Figure S41.** HMBC spectrum of **7** (600 MHz, DMSO-*d*_6_)


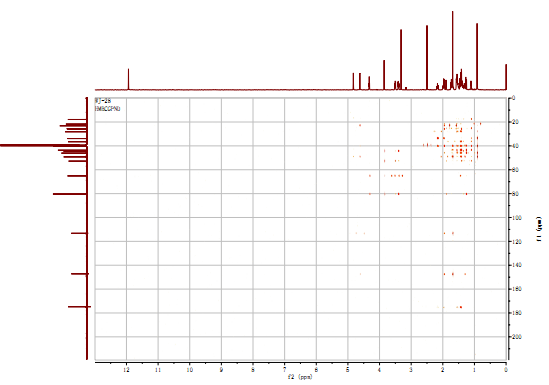


**Figure S42.** NOESY spectrum of **7** (600 MHz, DMSO-*d*_6_)


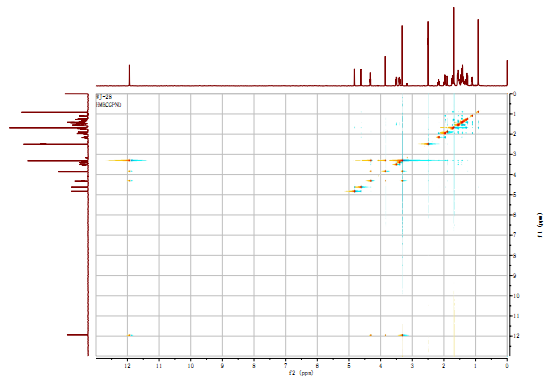


**Figure S43.** ^1^H NMR spectrum of **8** (600 MHz, CDCl_3_)

**Figure S44.** ^13^C NMR spectrum of **8** (150 MHz, CDCl_3_)

**Figure S45.** COSY spectrum of **8** (600 MHz, CDCl_3_)


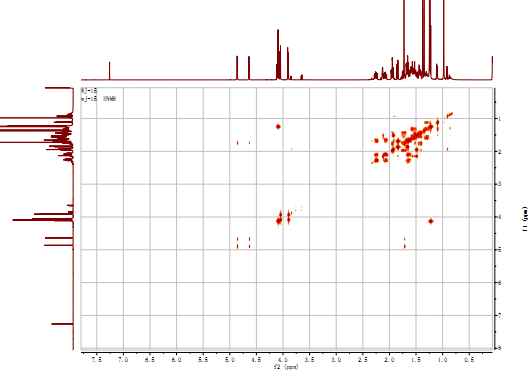


**Figure S46.** HMQC spectrum of **8** (600 MHz, CDCl_3_)


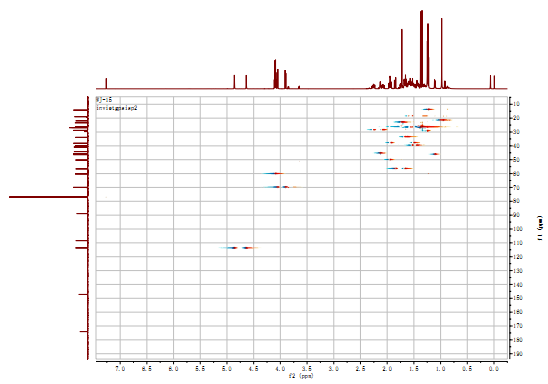


**Figure S47.** HMBC spectrum of **8** (600 MHz, CDCl_3_)


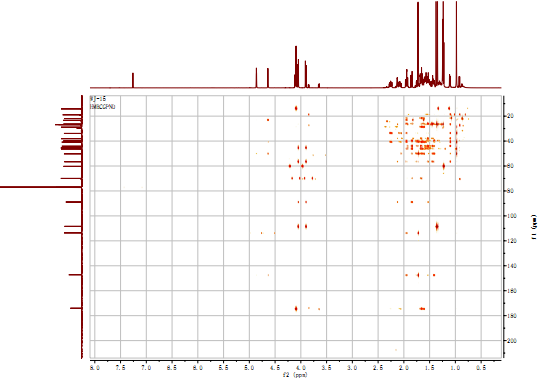


**Figure S48.** NOESY spectrum of **8** (600 MHz, CDCl_3_)


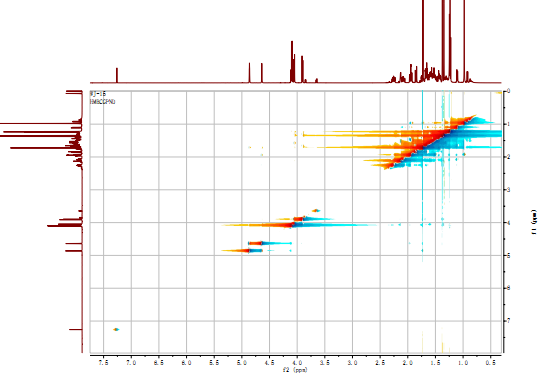


**Figure S49.** ^1^H NMR spectrum of **9** (600 MHz, DMSO-*d*_6_)

**Figure S50.** ^13^C NMR spectrum of **9** (150 MHz, DMSO-*d*_6_)

**Figure S51.** COSY spectrum of **9** (600 MHz, DMSO-*d*_6_)

**Figure S52.** HMQC spectrum of **9** (600 MHz, DMSO-*d*_6_)

**Figure S53.** HMBC spectrum of **9** (600 MHz, DMSO-*d*_6_)

**Figure S54.** NOESY spectrum of **9** (600 MHz, DMSO-*d*_6_)

**Figure S55.** ^1^H NMR spectrum of**10** (600 MHz, CD_3_OD)

**Figure S56.** ^13^C NMR spectrum of **10** (150 MHz, CD_3_OD)

**Figure S57.** COSY spectrum of **10** (600 MHz, CD_3_OD)

**Figure S58.** HMQC spectrum of **10** (600 MHz, CD_3_OD)

**Figure S59.** HMBC spectrum of **10** (600 MHz, CD_3_OD)

**Figure S60.** NOESY spectrum of **10** (600 MHz, CD_3_OD)

**Figure S61.** X-ray data of compound **1**

_audit_creation_method SHELXL-97

_chemical_name_systematic

;

?

;

_chemical_name_common ?

_chemical_melting_point ?

_chemical_formula_moiety 'C20 H32 O4, H2 O'

_chemical_formula_sum

'C20 H34 O5'

_chemical_formula_weight 354.47

_chemical_absolute_configuration ad

loop_

_atom_type_symbol

_atom_type_description

_atom_type_scat_dispersion_real

_atom_type_scat_dispersion_imag

_atom_type_scat_source

'C' 'C' 0.0181 0.0091

'International Tables Vol C Tables 4.2.6.8 and 6.1.1.4'

'H' 'H' 0.0000 0.0000

'International Tables Vol C Tables 4.2.6.8 and 6.1.1.4'

'N' 'N' 0.0311 0.0180

'International Tables Vol C Tables 4.2.6.8 and 6.1.1.4'

'O' 'O' 0.0492 0.0322

'International Tables Vol C Tables 4.2.6.8 and 6.1.1.4'

_symmetry_cell_setting 'orthorhombic'

_symmetry_space_group_name_H-M 'P 21 21 21'

_symmetry_space_group_name_Hall 'P 2ac 2ab'

loop_

_symmetry_equiv_pos_as_xyz

'x, y, z'

'-x+1/2, -y, z+1/2'

'x+1/2, -y+1/2, -z'

'-x, y+1/2, -z+1/2'

_cell_length_a 6.083(3)

_cell_length_b 11.417(3)

_cell_length_c 26.629(8)

_cell_angle_alpha 90.00

_cell_angle_beta 90.00

_cell_angle_gamma 90.00

_cell_volume 1849.4(12)

_cell_formula_units_Z 4

_cell_measurement_temperature 295(2)

_cell_measurement_reflns_used 2995

_cell_measurement_theta_min 6.32

_cell_measurement_theta_max 66.60

_exptl_crystal_description 'Prism'

_exptl_crystal_colour 'Colorless'

_exptl_crystal_size_max 0.52

_exptl_crystal_size_mid 0.14

_exptl_crystal_size_min 0.08

_exptl_crystal_density_meas ?

_exptl_crystal_density_diffrn 1.273

_exptl_crystal_density_method 'not measured'

_exptl_crystal_F_000 776

_exptl_absorpt_coefficient_mu 0.722

_exptl_absorpt_correction_type 'Multi-scan'

_exptl_absorpt_correction_T_max 0.956

_exptl_absorpt_correction_T_min 0.910

_exptl_absorpt_process_details ?

_exptl_special_details

;

?

;

_diffrn_ambient_temperature 295(2)

_diffrn_radiation_wavelength 1.54187

_diffrn_radiation_type CuK\a

_diffrn_radiation_source 'fine-focus sealed tube'

_diffrn_radiation_monochromator confocal

_diffrn_measurement_device_type 'MicroMax 002+'

_diffrn_measurement_method \f\k

_diffrn_detector_area_resol_mean 0

_diffrn_standards_number 0

_diffrn_standards_interval_count 0

_diffrn_standards_interval_time 0

_diffrn_standards_decay_% 0

_diffrn_reflns_number 7645

_diffrn_reflns_av_R_equivalents 0.0367

_diffrn_reflns_av_sigmaI/netI 0.0383

_diffrn_reflns_limit_h_min -5

_diffrn_reflns_limit_h_max 6

_diffrn_reflns_limit_k_min -13

_diffrn_reflns_limit_k_max 13

_diffrn_reflns_limit_l_min -31

_diffrn_reflns_limit_l_max 30

_diffrn_reflns_theta_min 6.32

_diffrn_reflns_theta_max 66.60

_reflns_number_total 3145

_reflns_number_gt 2995

_reflns_threshold_expression >2sigma(I)

_computing_data_collection 'CrystalClear (Rigaku Inc., 2008)'

_computing_cell_refinement 'CrystalClear (Rigaku Inc., 2008)'

_computing_data_reduction 'CrystalClear (Rigaku Inc., 2008)'

_computing_structure_solution 'SHELXS-97 (Sheldrick, 1990)'

_computing_structure_refinement 'SHELXL-97 (Sheldrick, 1997)'

_computing_molecular_graphics 'ORTEPII(Johnson,1976)andPLUTON(Spek,1990)'

_computing_publication_material 'SHELXL97(Sheldrick, 1997)'

_refine_special_details

;

Refinement of F^2^ against ALL reflections. The weighted R-factor wR and

goodness of fit S are based on F^2^, conventional R-factors R are based

on F, with F set to zero for negative F^2^. The threshold expression of

F^2^ > 2sigma(F^2^) is used only for calculating R-factors(gt) etc. and is

not relevant to the choice of reflections for refinement. R-factors based

on F^2^ are statistically about twice as large as those based on F, and R-

factors based on ALL data will be even larger.

;

_refine_ls_structure_factor_coef Fsqd

_refine_ls_matrix_type full

_refine_ls_weighting_scheme calc

_refine_ls_weighting_details

'calc w=1/[\s^2^(Fo^2^)+(0.0610P)^2^+0.1544P] where P=(Fo^2^+2Fc^2^)/3'

_atom_sites_solution_primary direct

_atom_sites_solution_secondary difmap

_atom_sites_solution_hydrogens geom

_refine_ls_hydrogen_treatment mixed

_refine_ls_extinction_method SHELXL

_refine_ls_extinction_coef 0.0070(10)

_refine_ls_extinction_expression

'Fc^*^=kFc[1+0.001xFc^2^\l^3^/sin(2\q)]^-1/4^'

_refine_ls_abs_structure_details

'Flack H D (1983), Acta Cryst. A39, 876-881'

_refine_ls_abs_structure_Flack -0.1(2)

_refine_ls_number_reflns 3145

_refine_ls_number_parameters 235

_refine_ls_number_restraints 2

_refine_ls_R_factor_all 0.0391

_refine_ls_R_factor_gt 0.0377

_refine_ls_wR_factor_ref 0.0996

_refine_ls_wR_factor_gt 0.0977

_refine_ls_goodness_of_fit_ref 1.049

_refine_ls_restrained_S_all 1.049

_refine_ls_shift/su_max 0.000

_refine_ls_shift/su_mean 0.000

loop_

_atom_site_label

_atom_site_type_symbol

_atom_site_fract_x

_atom_site_fract_y

_atom_site_fract_z

_atom_site_U_iso_or_equiv

_atom_site_adp_type

_atom_site_occupancy

_atom_site_symmetry_multiplicity

_atom_site_calc_flag

_atom_site_refinement_flags

_atom_site_disorder_assembly

_atom_site_disorder_group

O1 O 0.3322(3) -0.15055(13) 0.37025(5) 0.0588(4) Uani 1 1 d . . .

O2 O 0.7639(2) -0.20618(9) 0.44353(4) 0.0402(3) Uani 1 1 d . . .

H2A H 0.7941 -0.2035 0.4735 0.060 Uiso 1 1 calc R . .

O4 O 1.0175(4) 0.70321(14) 0.30353(5) 0.0728(5) Uani 1 1 d . . .

H4A H 1.0839 0.7361 0.3263 0.109 Uiso 1 1 calc R . .

O3 O 1.0294(2) 0.62089(10) 0.40168(5) 0.0442(3) Uani 1 1 d . . .

H3A H 0.9461 0.6708 0.4130 0.066 Uiso 1 1 calc R . .

C1 C 0.5879(3) 0.09160(15) 0.30967(6) 0.0393(4) Uani 1 1 d . . .

H1A H 0.4448 0.1290 0.3109 0.047 Uiso 1 1 calc R . .

H1B H 0.6519 0.1081 0.2771 0.047 Uiso 1 1 calc R . .

C2 C 0.5572(4) -0.04103(16) 0.31500(6) 0.0471(5) Uani 1 1 d . . .

H2B H 0.4529 -0.0675 0.2899 0.057 Uiso 1 1 calc R . .

H2C H 0.6964 -0.0794 0.3083 0.057 Uiso 1 1 calc R . .

C3 C 0.4766(3) -0.07793(14) 0.36611(6) 0.0374(4) Uani 1 1 d . . .

C4 C 0.5899(3) -0.02449(14) 0.41190(6) 0.0326(4) Uani 1 1 d . . .

C5 C 0.6362(3) 0.10856(13) 0.40215(5) 0.0302(4) Uani 1 1 d . . .

H5A H 0.4901 0.1446 0.4024 0.036 Uiso 1 1 calc R . .

C6 C 0.7578(4) 0.16874(14) 0.44564(6) 0.0379(4) Uani 1 1 d . . .

H6A H 0.7033 0.1392 0.4774 0.045 Uiso 1 1 calc R . .

H6B H 0.9137 0.1515 0.4436 0.045 Uiso 1 1 calc R . .

C7 C 0.7220(4) 0.30054(14) 0.44285(6) 0.0401(4) Uani 1 1 d . . .

H7A H 0.7988 0.3377 0.4705 0.048 Uiso 1 1 calc R . .

H7B H 0.5664 0.3170 0.4467 0.048 Uiso 1 1 calc R . .

C8 C 0.8024(3) 0.35333(13) 0.39355(5) 0.0306(4) Uani 1 1 d . . .

C9 C 0.7113(3) 0.28258(13) 0.34806(5) 0.0300(4) Uani 1 1 d . . .

H9A H 0.5524 0.2965 0.3488 0.036 Uiso 1 1 calc R . .

C10 C 0.7350(3) 0.14528(13) 0.35059(5) 0.0307(4) Uani 1 1 d . . .

C11 C 0.7898(3) 0.34045(15) 0.29852(6) 0.0392(4) Uani 1 1 d . . .

H11A H 0.7861 0.2816 0.2723 0.047 Uiso 1 1 calc R . .

H11B H 0.6847 0.4008 0.2895 0.047 Uiso 1 1 calc R . .

C12 C 1.0195(4) 0.39558(15) 0.29895(6) 0.0435(4) Uani 1 1 d . . .

H12A H 1.1273 0.3347 0.2926 0.052 Uiso 1 1 calc R . .

H12B H 1.0292 0.4517 0.2717 0.052 Uiso 1 1 calc R . .

C13 C 1.0780(3) 0.45726(14) 0.34803(7) 0.0386(4) Uani 1 1 d . . .

H13A H 1.2280 0.4884 0.3468 0.046 Uiso 1 1 calc R . .

C14 C 1.0538(3) 0.36863(14) 0.39089(6) 0.0360(4) Uani 1 1 d . . .

H14A H 1.1122 0.3993 0.4221 0.043 Uiso 1 1 calc R . .

H14B H 1.1266 0.2953 0.3830 0.043 Uiso 1 1 calc R . .

C15 C 0.7261(3) 0.48305(14) 0.38872(6) 0.0372(4) Uani 1 1 d . . .

H15A H 0.5951 0.4879 0.3681 0.045 Uiso 1 1 calc R . .

H15B H 0.6924 0.5150 0.4216 0.045 Uiso 1 1 calc R . .

C16 C 0.9151(3) 0.55225(14) 0.36435(6) 0.0363(4) Uani 1 1 d . . .

C17 C 0.9715(3) 0.10343(14) 0.34141(6) 0.0377(4) Uani 1 1 d . . .

H17A H 0.9766 0.0195 0.3432 0.057 Uiso 1 1 calc R . .

H17B H 1.0666 0.1361 0.3665 0.057 Uiso 1 1 calc R . .

H17C H 1.0190 0.1285 0.3088 0.057 Uiso 1 1 calc R . .

C18 C 0.4367(3) -0.03625(15) 0.45765(6) 0.0409(4) Uani 1 1 d . . .

H18A H 0.4075 -0.1176 0.4639 0.061 Uiso 1 1 calc R . .

H18B H 0.3010 0.0038 0.4510 0.061 Uiso 1 1 calc R . .

H18C H 0.5060 -0.0023 0.4866 0.061 Uiso 1 1 calc R . .

C19 C 0.8045(3) -0.09434(14) 0.42114(6) 0.0383(4) Uani 1 1 d . . .

H19A H 0.8800 -0.1055 0.3894 0.046 Uiso 1 1 calc R . .

H19B H 0.9002 -0.0493 0.4429 0.046 Uiso 1 1 calc R . .

C20 C 0.8408(4) 0.63762(17) 0.32381(7) 0.0521(5) Uani 1 1 d . . .

H20A H 0.7698 0.5943 0.2970 0.063 Uiso 1 1 calc R . .

H20B H 0.7333 0.6910 0.3380 0.063 Uiso 1 1 calc R . .

O1W O 0.3847(3) 0.65147(13) 0.46338(5) 0.0502(4) Uani 1 1 d D . .

H1WA H 0.481(5) 0.700(3) 0.4498(11) 0.099(11) Uiso 1 1 d D . .

H1WB H 0.294(5) 0.630(3) 0.4405(9) 0.085(10) Uiso 1 1 d D . .

loop_

_atom_site_aniso_label

_atom_site_aniso_U_11

_atom_site_aniso_U_22

_atom_site_aniso_U_33

_atom_site_aniso_U_23

_atom_site_aniso_U_13

_atom_site_aniso_U_12

O1 0.0633(10) 0.0526(8) 0.0605(8) -0.0051(6) -0.0030(7) -0.0270(8)

O2 0.0506(8) 0.0294(5) 0.0406(6) 0.0029(4) -0.0027(6) -0.0006(6)

O4 0.1092(15) 0.0569(8) 0.0524(7) 0.0172(7) 0.0023(9) -0.0284(11)

O3 0.0487(8) 0.0340(6) 0.0498(6) -0.0074(5) -0.0034(6) -0.0060(6)

C1 0.0470(11) 0.0391(9) 0.0319(7) -0.0023(6) -0.0044(8) -0.0040(8)

C2 0.0632(14) 0.0418(9) 0.0364(8) -0.0092(7) -0.0054(9) -0.0153(10)

C3 0.0377(10) 0.0299(8) 0.0445(8) -0.0025(6) -0.0019(8) -0.0014(8)

C4 0.0340(9) 0.0292(7) 0.0347(7) -0.0018(6) 0.0019(7) -0.0021(7)

C5 0.0329(9) 0.0278(7) 0.0301(7) -0.0015(6) -0.0008(7) 0.0005(6)

C6 0.0527(11) 0.0337(8) 0.0272(7) 0.0001(6) -0.0013(8) -0.0069(8)

C7 0.0565(12) 0.0321(8) 0.0316(7) -0.0082(6) 0.0066(8) -0.0075(8)

C8 0.0343(9) 0.0268(7) 0.0308(7) -0.0024(6) 0.0011(7) -0.0002(7)

C9 0.0295(9) 0.0296(8) 0.0309(7) -0.0009(6) -0.0007(7) 0.0001(7)

C10 0.0339(9) 0.0293(7) 0.0289(7) -0.0028(5) -0.0017(7) 0.0000(7)

C11 0.0544(12) 0.0333(8) 0.0300(7) 0.0010(6) -0.0025(8) -0.0016(8)

C12 0.0537(12) 0.0387(8) 0.0381(8) -0.0006(6) 0.0145(9) -0.0018(9)

C13 0.0349(10) 0.0333(8) 0.0477(9) 0.0005(7) 0.0052(8) -0.0054(8)

C14 0.0377(10) 0.0306(7) 0.0398(8) -0.0027(6) -0.0046(8) 0.0018(7)

C15 0.0367(10) 0.0308(8) 0.0440(8) -0.0046(6) 0.0034(8) 0.0023(7)

C16 0.0430(11) 0.0299(8) 0.0359(7) -0.0022(6) 0.0018(7) -0.0036(7)

C17 0.0384(10) 0.0321(8) 0.0426(8) -0.0025(6) 0.0051(8) 0.0021(8)

C18 0.0442(11) 0.0354(8) 0.0430(8) -0.0005(7) 0.0097(8) -0.0024(9)

C19 0.0406(11) 0.0307(8) 0.0437(8) 0.0036(6) 0.0012(8) -0.0019(8)

C20 0.0709(15) 0.0359(9) 0.0496(9) 0.0071(8) -0.0028(10) -0.0009(10)

O1W 0.0566(10) 0.0507(7) 0.0432(7) -0.0006(6) 0.0052(7) -0.0087(7)

_geom_special_details

;

All esds (except the esd in the dihedral angle between two l.s. planes)

are estimated using the full covariance matrix. The cell esds are taken

into account individually in the estimation of esds in distances, angles

and torsion angles; correlations between esds in cell parameters are only

used when they are defined by crystal symmetry. An approximate (isotropic)

treatment of cell esds is used for estimating esds involving l.s. planes.

**Figure S62.** HR-MS of **1-10**

HR-MS of compound **1**


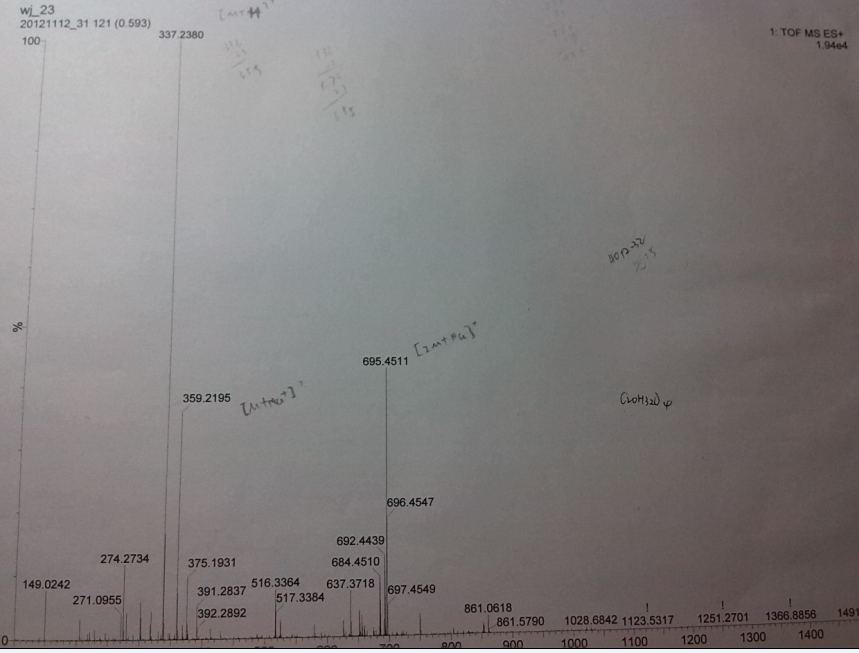


HR-MS of compound **2**


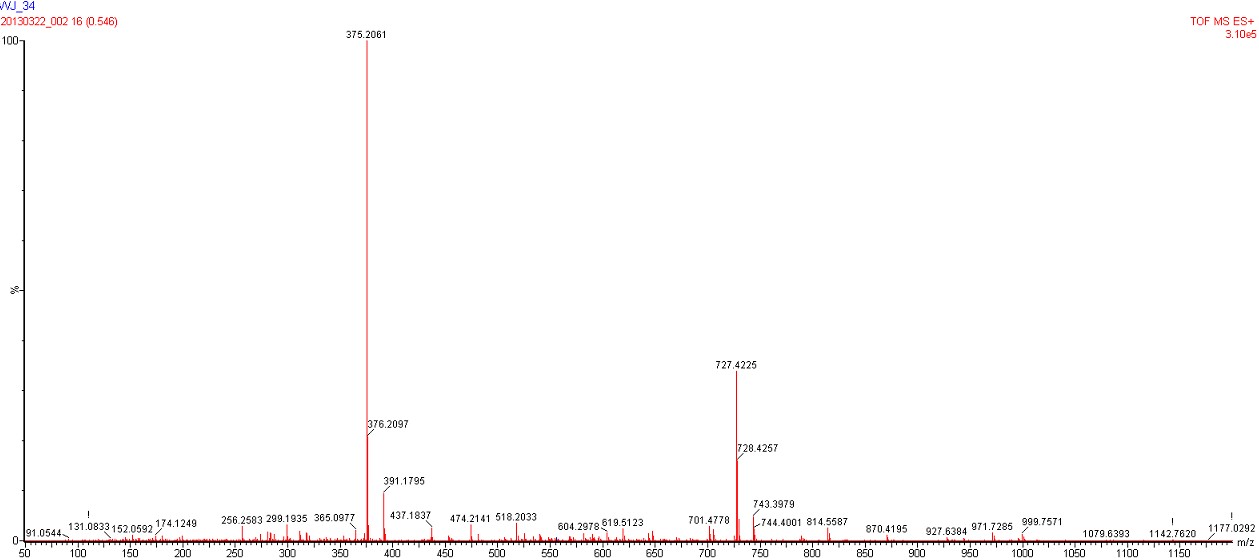


HR-MS of compound **3**

HR-MS of compound **4**

HR-MS of compound **5**

HR-MS of compound **6**

HR-MS of compound **7**


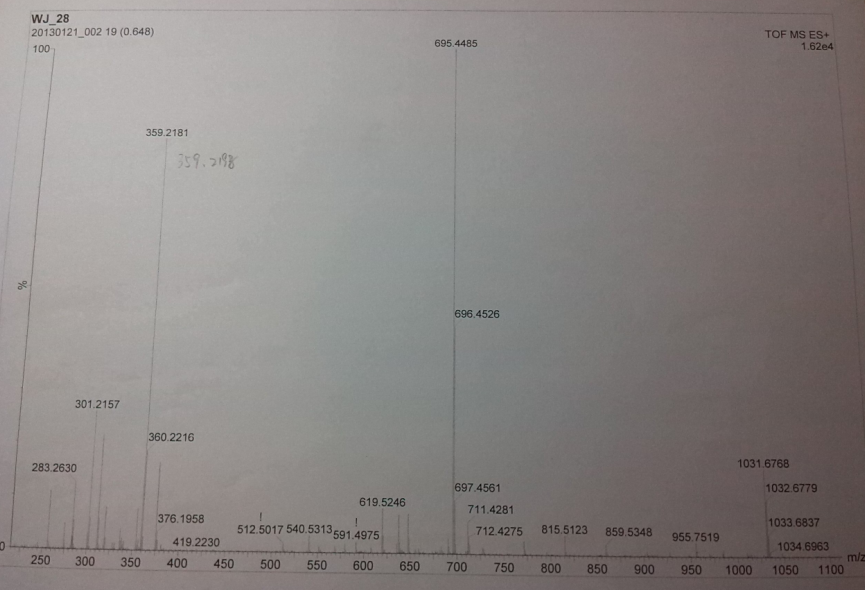


HR-MS of compound **8**


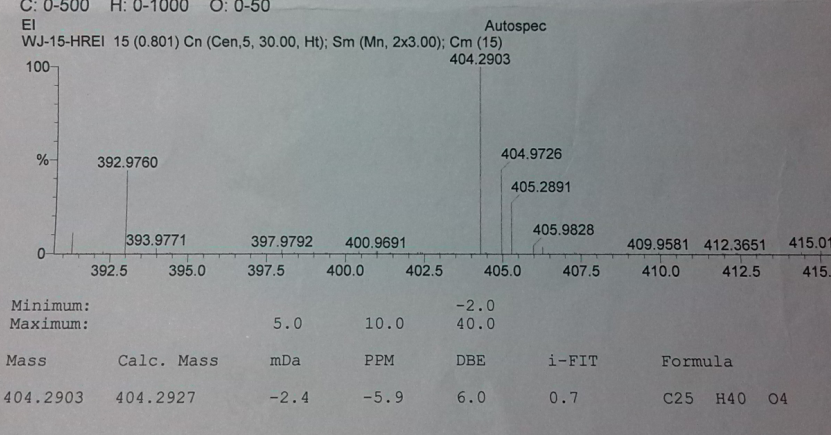


HR-MS of compound **9**

HR-MS of compound **10**


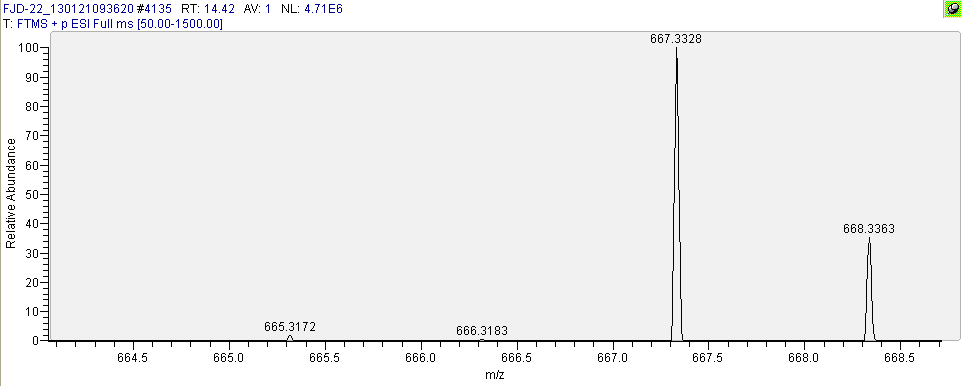


**Figure S63**  CD spectra of **1-10**

CD spectra of **1** CD spectra of **2**


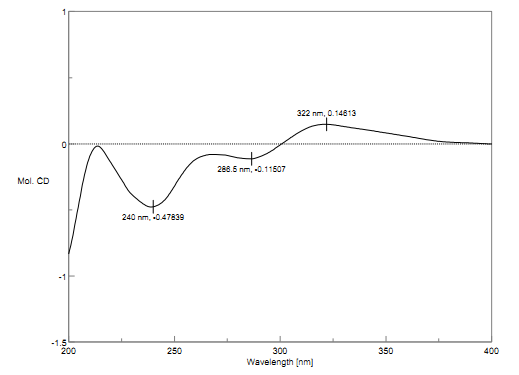

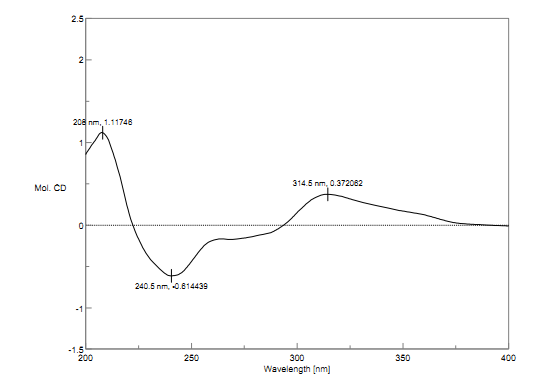


CD spectra of **3-6**

**
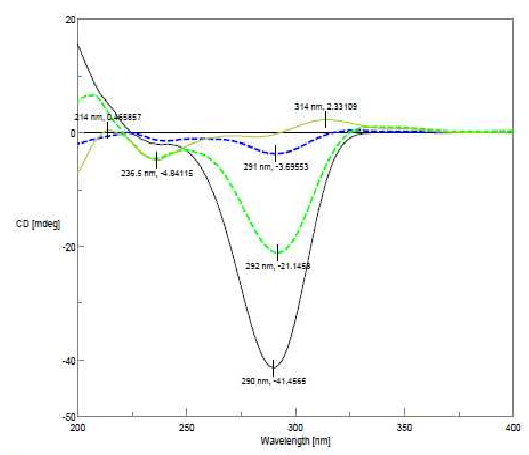
**

Compund **3**

Compund **4**

Compund **5**

Compund **6**

CD spectra of **7** CD spectra of **8**


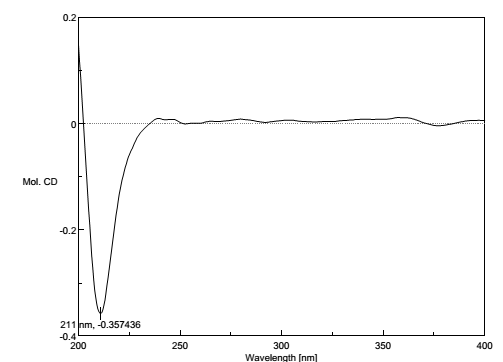

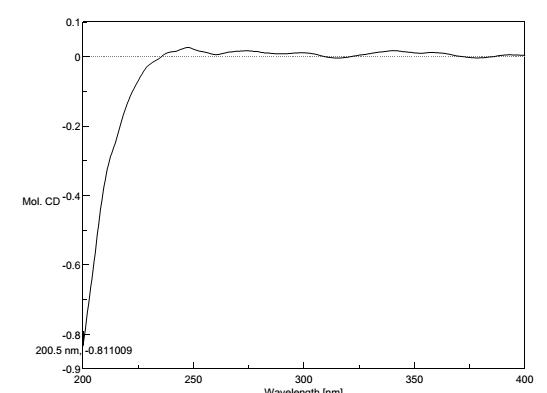


CD spectra of **9** CD spectra of **10**


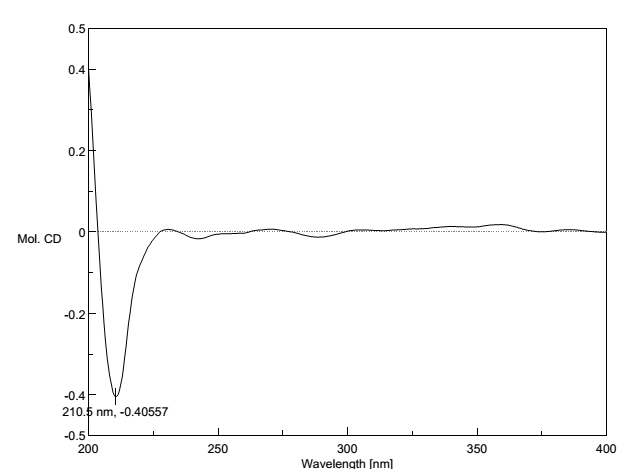

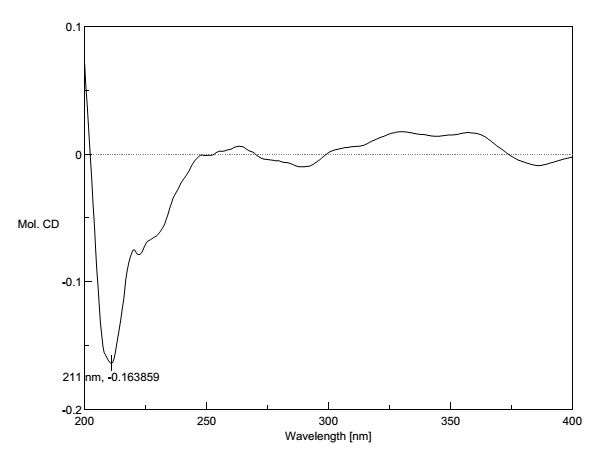

Supplement: Supplementary Information [file srep30560-s1.doc]
